# Construction of *Agropyron* Gaertn. genetic linkage maps using a wheat 660K SNP array reveals a homoeologous relationship with the wheat genome

**DOI:** 10.1111/pbi.12831

**Published:** 2017-10-16

**Authors:** Shenghui Zhou, Jinpeng Zhang, Yonghe Che, Weihua Liu, Yuqing Lu, Xinming Yang, Xiuquan Li, Jizeng Jia, Xu Liu, Lihui Li

**Affiliations:** ^1^ National Key Facility for Crop Gene Resources and Genetic Improvement Institute of Crop Sciences Chinese Academy of Agricultural Sciences Beijing China; ^2^ Department of Life Science and Technology Hebei Normal University of Science and Technology Qinhuangdao Hebei China

**Keywords:** wheat, wild relatives, SNP array, genetic linkage maps, homoeologous relationship, derivatives

## Abstract

*Agropyron* Gaertn. (P genome) is a wild relative of wheat that harbours many genetic variations that could be used to increase the genetic diversity of wheat. To agronomically transfer important genes from the P genome to a wheat chromosome by induced homoeologous pairing and recombination, it is necessary to determine the chromosomal relationships between *Agropyron* and wheat. Here, we report using the wheat 660K single nucleotide polymorphism (SNP) array to genotype a segregating *Agropyron* F_1_ population derived from an interspecific cross between two cross‐pollinated diploid collections ‘Z1842’ [*A. cristatum* (L.) Beauv.] (male parent) and ‘Z2098’ [*A. mongolicum* Keng] (female parent) and 35 wheat–*A. cristatum* addition/substitution lines. Genetic linkage maps were constructed using 913 SNP markers distributed among seven linkage groups spanning 839.7 cM. The average distance between adjacent markers was 1.8 cM. The maps identified the homoeologous relationship between the P genome and wheat and revealed that the P and wheat genomes are collinear and relatively conserved. In addition, obvious rearrangements and introgression spread were observed throughout the P genome compared with the wheat genome. Combined with genotyping data, the complete set of wheat–*A. cristatum* addition/substitution lines was characterized according to their homoeologous relationships. In this study, the homoeologous relationship between the P genome and wheat was identified using genetic linkage maps, and the detection mean for wheat–*A. cristatum* introgressions might significantly accelerate the introgression of genetic variation from *Agropyron* into wheat for exploitation in wheat improvement programmes.

## Introduction

Hexaploid bread wheat (*Triticum aestivum* L., 2*n* = 6*x* = 42, genomes AABBDD) is widely planted worldwide and is an important food source. However, the diversity of modern cultivated wheat has been restricted by selection for specific agronomically important traits during wheat domestication and improvement (Charmet, 2011; Khoury *et al*., 2014), limiting further wheat improvement (White *et al*., 2008). The hybridization of wheat with wild relatives, which are an untapped reservoir of substantial genetic variation for many agronomically important traits (Friebe *et al*., 1996; Jauhar and Chibbar, 1999; Qi *et al*., 2007; Schneider *et al*., 2008), has been used in numerous wheat breeding programmes to introduce novel diversity into the bread wheat gene pool and is called alien introgression (Armstead *et al*., 2006; Chen *et al*., 2012b; Molnár‐Láng *et al*., 2014; Zamir, 2001).

*Agropyron* Gaertn., a genus of wild relatives of wheat, is not only a type of pasture grass but also an excellent wild germplasm for wheat breeding (Ford‐Lloyd *et al*., 2011). *Agropyron* is distributed on the temperate‐frigid grassland and sand land of Eurasia and includes approximately 10–15 species, including the five most common members: *A. mongolicum*,* A. cristatum*,* A. cristatum* ssp. imbricatum, *A. cristatum* ssp. pectinatum and *A. fragile* (Dewey, 1984). *Agropyron* (P genome) encodes potentially valuable traits for wheat improvement, such as disease resistance, abiotic and biotic stress tolerance and high yield (Dong *et al*., 1992). In the early 1990s, the introduction of desirable genes from *A. cristatum* accession Z559 into common wheat cv. Fukuhokomugi (Fukuho) was achieved by intergeneric hybridization (Li *et al*., 1998). Subsequently, a set of additional wheat–*A. cristatum* lines and disomic substitution lines were produced and characterized (Han *et al*., 2017; Wu *et al*., 2006), some of which will be valuable for future wheat breeding as novel germplasms (Chen *et al*., 2012a; Wu *et al*., 2006). For example, the 6P disomic addition line 4844 shows high yield characteristics for the number of florets and kernels per spike compared with those of its wheat parent (Li *et al*., 1998; Wu *et al*., 2006), and resistance to powdery mildew and leaf rust was transferred from *A. cristatum* to common wheat (Li *et al*., 2016).

Although there have been some notable successes transferring valuable genetic variation from *A. cristatum*, only a fraction of this plant's full potential has been exploited in breeding. Understanding the homoeologous relationships and degree of collinearity between the chromosomes of the species of interest is important to select an optimal alien chromosome transfer strategy that will allow the introgression of only a small portion of the alien chromosome, without transferring undesirable traits, and compensate for the replaced wheat chromatin (Danilova *et al*., 2014; Qi *et al*., 2007). Several approaches have been used to study the homoeology between hexaploid wheat and wild relative chromosomes, such as gametophytic compensation tests (Dvořák, 1980; Friebe *et al*., 1993; Sears, 1952), chromosome pairing tests (Yang *et al*., 1996) and fluorescence in situ hybridization (FISH) analyses (Danilova *et al*., 2012, 2014). Creating a comparative genetic linkage map analysis of alien chromosomes with wheat is an approach that employs molecular markers to develop and analyse segregating populations (Danilova *et al*., 2014). Recently, Zhang *et al*. (2015c) constructed a high‐density genetic map for *Agropyron* Gaertn. based on single nucleotide polymorphisms (SNPs) from specific‐locus amplified fragment sequencing (SLAF‐seq). However, because these SNPs were essentially P genome‐specific markers from noncoding DNA sequences which were highly differentiated among species, the homoeologous relationship between the P genome and hexaploid wheat chromosomes could not be determined.

With the development of a high‐density iSelect array (Wang *et al*., 2014) and the use of kompetitive allele‐specific PCR (KASP) assays (Allen *et al*., 2011), SNPs are increasingly being employed in the genotyping of wheat and its wild relatives as well as their introgression lines. Winfield *et al*. (2015) used a wheat NimbleGen array (Winfield *et al*., 2012) to direct the capture and targeted resequencing of the wheat exome and identified a large number of SNPs from 43 bread wheat accessions and wheat relatives. Moreover, 218 genome‐wide wheat/*Ambylopyrum muticum* introgressions were detected and characterized using these SNP markers, and seven linkage groups of *Am. muticum* were constructed to determine the syntenic relationships between the wild relative and hexaploid wheat (King *et al*., 2017). Therefore, the array developed using these SNPs can be employed to characterize wheat‐related species and would provide the wheat community with a valuable resource for the characterization and breeding of hexaploid and tetraploid wheat. In addition, an ultra‐high‐throughput 660K wheat array based on Affymetrix® Axiom® and Illumina Infinium technology including more than 630K SNPs developed from transcriptome and genome sequencing has been announced. This array is highly efficient with a wide range of potential applications (http://wheat.pw.usda.gov/ggpages/topics/Wheat660_SNP_array_developed_by_CAAS.pdf). Nevertheless, because there is high sequence similarity between wheat and wild relatives for transcriptome sequences (Krasileva *et al*., 2013; Zhang *et al*., 2015b), the wheat 660K SNP array also serves as an invaluable resource for genotyping wild relatives of wheat.

In this study, a segregating *Agropyron* F_1_ population derived from an interspecific cross was used as the cross‐pollinated (CP) population to construct a genetic linkage map. The wheat 660K SNP array generated genotype data to reveal the homoeologous relationships, chromosome rearrangements and degree of collinearity between the P genome and wheat. In addition, we provided the first report on the production of a complete set of wheat–*A. cristatum* addition lines. Combined with the wheat 660K SNP genotyping array and the analysis of homoeologous relationships, an efficient high‐throughput screening method was developed to identify and characterize addition/substitution lines.

## Results

### Genotyping and SNP filtration of the CP population

The Axiom Wheat 660K Genotyping Array was used to genotype the *Agropyron* F_1_ CP population (two parents and 119 progenies). The most basic postgenotyping filter is based on the sample quality control call rate. The minimum, maximum and mean of the call rate for the 121 individuals were 74.6%, 80.6% and 77.4%, respectively (Figure S1). The wheat 660K assay interrogated 630 517 markers, of which 59 153 markers had a 100% call rate and 43.9% (276 961) markers had call rates above 80% (Figure S1). The relatively low call rate primarily reflects the genomic differences between *Agropyron* Gaertn and wheat. However, the wheat 660K genotyping array still provides a robust resource for genome‐wide, high‐density SNP genotyping and population genetic analyses of wild wheat relatives such as *Agropyron* Gaertn.

A full description of the genotyping and categorization is provided in the Experimental procedures. A total of 18 192 SNP markers, including 4 196 codominant and polymorphic Poly High Resolution SNP markers and 133 996 polymorphic and dominant No Minor Homozygote SNP markers in the CP population, were used for genetic mapping (Table S1). Approximately 97.1% of SNP markers contained in Mono High Resolution, Call Rate Below Threshold, Off‐Target Variant and other categories were removed because of one or more cluster properties below the threshold. These results indicated that only 2.9% of wheat SNP markers were present in the *Agropyron* F_1_ CP population.

The segregation patterns (hk × hk, lm × ll and nn × np) were appropriate for the CP population. Of the 18 192 Poly High Resolution and No Minor Homozygote SNP markers, 2 694 SNP markers were categorized into these three types. After removing markers with high segregation distortion, a total of 1 544 SNP markers, which included 735, 390 and 419 SNP markers for hk × hk, lm × ll and nn × np segregation patterns, respectively, remained for subsequent genetic mapping (Table S2).

### Construction of genetic linkage maps for the *Agropyron* CP population

**Figure 1:**
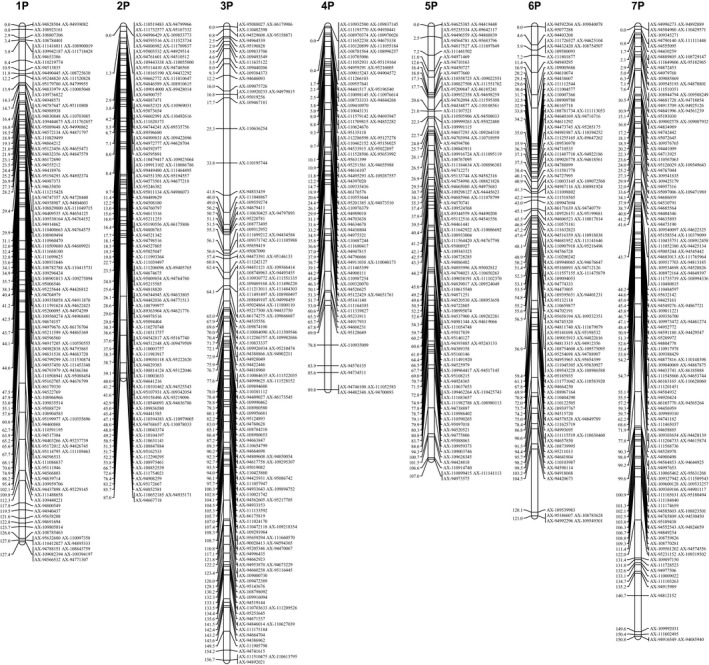
Integrated genetic linkage map for *Agropyron*.

After linkage analysis, 559 SNP markers for the male map (Table S3 and Figure S2), 521 SNP markers for the female map (Table S3 and Figure S3) and 913 SNP markers for the integrated map (Table S4, Figure 1) were located in the genetic map, all of which were assigned to seven linkage groups (LGs). Using BLASTN, uniquely aligned SNP tags in the genome of hexaploid wheat were used to assign LGs to chromosomes (Table S5). In the integrated map, a total of 78.5% of SNPs in LG1, 65.1% of SNPs in LG2, 78.2% of SNPs in LG3, 78.0% of SNPs in LG5, 73.6% of SNPs in LG6 and 66.5% of SNPs in LG7 were distributed in 1, 2, 3, 5, 6 and 7 homoeologous groups in the wheat genome, respectively, showing a substantial corresponding relationship between LG1 and 1P, LG2 and 2P, LG3 and 3P, LG5 and 5P, LG6 and 6P and LG7 and 7P (Tables S4 and S6). However, most SNP markers on LG4 were simultaneously distributed in homoeologous groups 2 and 4 in the wheat genome. Because of a previous linkage group LG2 for 2P and the correct verification for 4P addition lines (mentioned below, Table 1), LG4 was regarded as 4P (Table S4). The distribution in both homoeologous groups 2 and 4 in the wheat genome for LG4 might reflect chromosome structural rearrangements between 2P and 4P in *Agropyron*.

**Table 1:** Characterization of wheat–*A. cristatum* addition/substitution lines

Accession number	Chromosome composition	Additional alien chromosome	Missing chromosome	Identification result	References
10521‐2	42W + 2P	1P		1P addition line	
II‐3‐1A‐1	42W + 4P	1P and 2P		1P and 2P addition line	
II‐3‐1	40W + 4P	1P and 2P	1A	2P addition and 1P/1A substitution line	Pan *et al*. (2017)
II‐4	42W + 4P	1P and 2P		1P and 2P addition line	
2‐57‐1	42W + 2P	2P		2P addition line	
2‐72	42W + 2P	2P		2P addition line	
II‐29‐1	42W + 2P	2P		2P addition line	
II‐9‐3	42W + 2P	2P		2P addition line	Han *et al*. (2017)
II‐8‐1	42W + 2P	2P		2P addition line	Han *et al*. (2017)
II‐7‐1	42W + 2P	2P		2P addition line	Han *et al*. (2017)
7365	40W + 2P	3P	3B	3P/3B substitution line	
4‐11	42W + 2P	4P		4P addition line	
4‐12	42W + 2P	4P		4P addition line	
4‐6	42W + 2P	4P		4P addition line	
4‐7	42W + 2P	4P		4P addition line	
4‐8	42W + 2P	4P		4P addition line	
II‐21‐2	42W + 2P	4P		4P addition line	Han *et al*. (2017)
II‐21‐6	42W + 2P	4P		4P addition line	Han *et al*. (2017)
35524	42W + 2P	5P		5P addition line	
II‐11‐1	42W + 4P	2P and 5P		2P and 5P addition line	
5113‐2	42W + 2P	6P		6P addition line	Han *et al*. (2017)
5114‐3	42W + 2P	6P		6P addition line	Han *et al*. (2017)
II‐30‐5	42W + 2P	6P		6P addition line	
4844‐12	42W + 2P	6P		6P addition line	Han *et al*. (2017)
4844‐8	40W + 2P	6P	6D	6P/6D substitution line	Wu *et al*. (2006)
II‐1‐1	42W + 2P	7P		7P addition line	
7‐49	42W + 2P	7P		7P addition line	
7‐64	40W + 2P	7P	7D	7P/7D substitution line	
7‐65	40W + 2P	7P	7D	7P/7D substitution line	
7‐7	40W + 2P	7P	7A	7P/7A substitution line	
5038	42W + 2P	7P		7P addition line	Han *et al*. (2017)
5043	42W + 2P	7P		7P additioin line	Han *et al*. (2017)
II‐1‐3	42W + 2P	7P		7P additioin line	
II‐5‐1	42W + 2P	7P		7P additioin line	Han *et al*. (2017)
II‐26‐1	42W + 2P	7P		7P additioin line	Han *et al*. (2017)

[Correction added on 3 January 2018, after first online publication: In table 1, the chromosome composition for accession number: 10521‐2 was previously wrong. This has been corrected in this current version.]

The basic characteristics of the seven integrated genetic maps are shown in Table S4. Considering the size of all genetic maps, marker coverage totalled 839.7 cM for the genetic maps. The final map contained 471 loci with an average distance of 1.8 cM between adjacent loci (Table S4). The positions of all SNP markers on every chromosome are shown in Table S5. On average, one chromosome contained 130.4 SNP markers and 67.3 loci, spanning an average of 120.0 cM. The genetic distance of the maps ranged from 87.0 cM (2P chromosome with 130 markers and an average distance of 1.9 cM between adjacent loci) to 156.7 cM (3P chromosome with 143 markers and an average distance of 1.8 cM between adjacent loci). The degree of linkage between markers was reflected as ‘Gap ≤ 5’ and ranged from 95.4% (4P chromosome) to 96.9% (2P chromosome) with an average value of 96.0%. The largest gap on this map was 15.6 cM in the 6P chromosomal genetic map.

### Syntenic relationship between wheat and *Agropyron* Gaertn.

**Figure 2:**
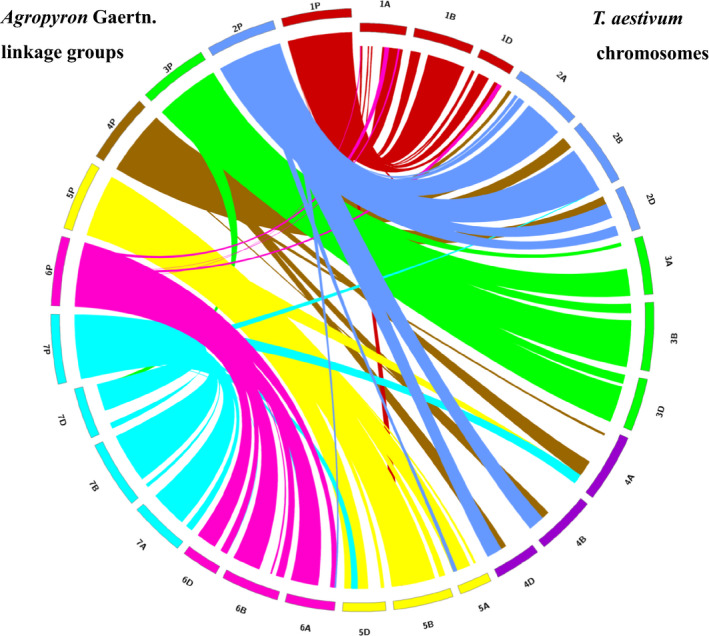
Synteny of *Agropyron* (genetic position in cM) with hexaploid wheat (physical position in Mb).

The parental map and integrated map make it possible to perform a comparison of collinearity between the two parents. The genetic maps of the two parents showed good collinearity with the integrated map, except for a few obvious rearrangements on chromosomes 2P, 4P and 7P (Figure S4). Therefore, the male and female genetic linkage maps were compared with wheat genome to identify the male‐ and female‐specific syntenic relationships with wheat, respectively (Figures S5 and S6). Figure 2 shows the degree of collinearity between the integrated P chromosomes and each of the three wheat genomes, with large ‘ribbons’ showing significant collinearity. Good collinearity was observed between chromosomes 1P, 2P, 3P, 4P, 5P, 6P and 7P, and LG 1, 2, 3, 4, 5, 6 and 7 of hexaploid wheat, respectively (Figure 2), which further confirmed the corresponding relationship between LGs and P chromosomes. However, the P genome showed obvious rearrangements in different chromosomes compared with the wheat chromosome. Segments of chromosome 4P were also observed on the short arm of group 2 in hexaploid wheat, whereas chromosome 2P and 7P segments were duplicated in the telomeric region of the long arm of group 4 in hexaploid wheat (Figure 2). In addition, small chromosome fragment rearrangements were obvious when narrow ‘ribbons’ were employed for cross‐mapping to noncollinear positions on the wheat chromosomes, such as some fragments of chromosome 6P corresponding to chromosomes 1A and 1D. Thus, this analysis indicated that the P genome underwent several chromosomal rearrangements compared with wheat chromosomes.

### Identification of addition/substitution lines

**Figure 3:**
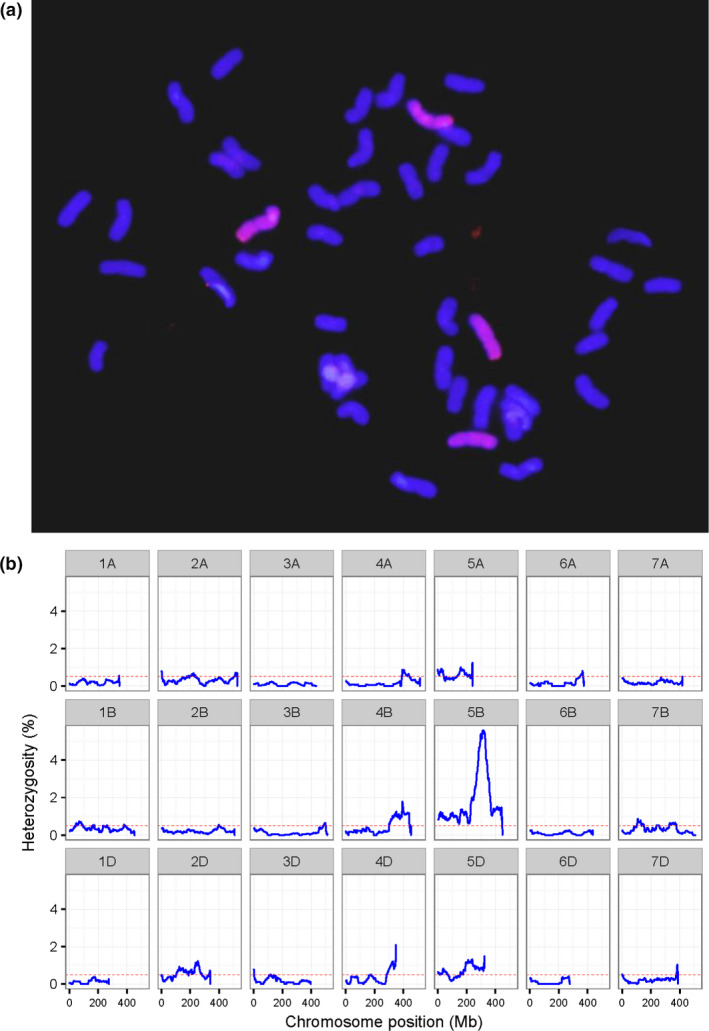
Characterization of the double disomic addition line II‐11‐1. (a) GISH analysis showing chromosome composition with *A. cristatum* genomic DNA as the probe and Fukuho DNA as blocker. (b) Distribution of heterozygosity in wheat genome. Here, heterozygosity is the ratio of heterozygous SNPs to all SNPs, which were counted in sliding windows of 50 Mb with a step of 1 Mb and plotted along the chromosome.

A total of 35 addition/substitution lines, namely 26 disomic addition lines, three double disomic addition lines, five substitution lines and one double disomic addition and substitution line, were identified using genomic in situ hybridization (GISH) from derivatives of hybridizations between wheat and *A. cristatum* (Table 1, Figure 3a and Figure S7). To identify the *A. cristatum* chromosomes incorporated into all 35 addition/substitution lines, the ratio of heterozygous genotypes on each of the wheat chromosomes for each addition/substitution line was plotted (Figure 3b and Figure S8). Then, according to the homoeologous relationships between P LGs and wheat chromosomes, the P chromosomes that were homoeologous to the most heterozygous wheat chromosomes were regarded as additional alien chromosomes for disomic addition lines, and the two P chromosomes corresponding to wheat chromosomes showing the highest heterozygosity were counted as additional chromosomes for double disomic addition lines. For example, II‐11‐1 was a double disomic addition (chromosome composition 42W + 4P) identified via GISH analysis (Figure 3a). The genotyping results for II‐11‐1 showed that its heterozygous SNPs were mainly distributed on chromosomes 2D, 5A, 5B, 5D and the long arms of 4B and 4D (Figure 3b). The syntenic relationships indicated that these highly heterozygous chromosomes were homoeologous to chromosomes 2P and 5P (Figure 2); therefore, the additional alien chromosomes in the II‐11‐1 double disomic addition line were 2P and 5P (Table 1). Furthermore, 1 1P (10521‐2), 6 2P (2‐57‐1, 2‐72, II‐29‐1, II‐9‐3, II‐7‐1 and II‐8‐1), 7 4P (4‐11, 4‐12, 4‐6, 4‐7, 4‐8, II‐21‐2 and II‐21‐6), 1 5P (35524), 4 6P (5113‐2, 5114‐3, II‐30‐5 and 4844‐112) and 7 7P (II‐1‐1, 7‐49, 5038, 5043, II‐1‐3, II‐5‐1 and II‐26‐1) disomic addition lines and 2 1P‐2P (II‐3‐1A‐1 and II‐4‐2) double disomic addition lines were identified in the same way (Table 1). The genotyping miss rate of wheat for each chromosome was also calculated to confirm the missing chromosomes in the substitution lines (Table S7). Subsequently, 1 3P/3B (7365), 1 6P/6D (4844‐8), 2 7P/7D (7‐64 and 7‐65) and 1 7P/7A (7‐7) substitution lines as well as 1 2P addition and 1P/1A substitution line (II‐3‐1) were identified in this study (Table 1). We obtained and verified a complete set of wheat–*A. cristatum* derivatives involving the introgression of all P chromosomes.

## Discussion

To broaden the genetic variation in wheat, desirable genes from *A. cristatum* (P genome) were successfully introduced into the common wheat variety Fukuho using intergeneric hybridization (Li *et al*., 1998). However, the progress in P genomic *A. cristatum* studies lags far behind the production and application of wheat–*A. cristatum* derivatives, limiting our further understanding of the genetic effects of alien fragments/genes on agronomic traits and the application of these derivative lines to wheat breeding projects. Thus far, the P genome of *Agropyron* has not been sequenced because of its large size. Therefore, the construction of a high‐density and high‐quality genetic map is critically important to fully exploit the wheat–*A. cristatum* novel germplasm until whole‐genome sequencing has been achieved. In this study, a genetic linkage map was constructed using a segregating *Agropyron* F_1_ population derived from an interspecific cross as the CP population using the wheat 660K SNP genotyping array. The study provided useful information and showed three major features.

### Construction of genetic linkage maps for *Agropyron* based on the wheat 660K SNP array

In this study, we constructed a genetic linkage map based on the wheat 660K SNP array to determine the highest coverage density and degree (reflected by ‘Gap ≤ 5’) linkage map of *Agropyron*. The map spanned a total of 839.7 cM, with an average of 130.4 markers and 67.3 loci per LG and an average distance of 1.8 cM between adjacent loci. Previously, a map was ordered in seven LGs using 152 AFLP and 23 RAPD markers using fewer molecular markers (Yu *et al*., 2012). Another map was constructed using 1 023 SNPs developed from SLAF‐seq (Zhang *et al*., 2015c). However, the SNPs from SLAF‐seq were derived from genome noncoding region sequencing and exhibit low identity compared with the wheat genome. Hence, the low coverage and backward P genome‐specific molecular markers limited the further application of these genetic maps. The successful genotyping of *Agropyron* using the wheat 660K SNP array indicated that genotyping different genera to construct the genetic maps using the available SNP array is possible. In addition, this genetic map can be used to anchor the scaffolds of the P genome and to study comparative Triticeae genomics.

### Comparative analysis of the homoeologous relationships between P genome chromosomes and wheat genome chromosomes

This study provided the first knowledge of the homoeologous relationships between *Agropyron* and common wheat based on the wheat 660K array platform. In the seven LGs constructed for *Agropyron* in this study, 78.5% of SNPs on LG1, 65.1% of SNPs on LG2, 78.2% of SNPs on LG3, 78.0% of SNPs on LG5, 73.6% of SNPs on LG6 and 66.5% of SNPs on LG7 were assigned to 1, 2, 3, 5, 6 and 7 homoeologous groups (A/B/D) in the wheat genome, respectively (Table S4), showing the relationships between LG1 and 1A/B/D, LG2 and 2A/B/D, LG3 and 3A/B/D, LG5 and 5A/B/D, LG6 and 6A/B/D and LG7 and 7A/B/D. Therefore, the six LGs, LG1, LG2, LG3, LG5, LG6 and LG7, were assigned to 1P, 2P, 3P, 5P, 6P and 7P accordingly. These groups performed well in synteny to wheat homoeologs, which were also identified using polymorphic SSR and EST‐SSR markers for six wheat–*A. cristatum* 6P addition lines (Han *et al*., 2014) and through BLAT alignment of *Agropyron* SLAF marker sequences with the draft genome assemblies of wheat (Zhang *et al*., 2015c). However, most SNP markers on LG4 were simultaneously located in 2A/B/D and 4A/B/D in the wheat genome. The correct verification for 4P addition lines using this linkage map (Table 1) and the previously identified linkage group LG2 for 2P indicated that LG4 should be assigned to 4P. This result reflects an active interchromosomal translocation between 2P and 4P in *Agropyron*. Well‐compensating translocations or introgressions produced via recombination between alien chromosomal regions and homoeologous wheat chromosomes have been found to be beneficial for wheat improvement, such as *Lr24*/*Sr24*,* Sr26* from *Thinopyrum elongatum*,* Sr36*/*Pm6* from *Triticum timopheevii*,* Lr26*/*Sr31*/*Yr9*/*Pm8* from the translocation line T1BL·1R#1S, *Gb2*/*Pm17* from T1AL·1R#2S of *Secale cereale* and *Yr17*/*Lr37*/*Sr38*/*Cre5* from *Aegilops ventricosa* (Friebe *et al*., 1996; Gill *et al*., 2006). In this study, the collinearity analyses not only confirmed the corresponding relationship between LGs and P chromosomes but also showed that the P genome had undergone obvious rearrangements in different chromosomes compared with the wheat chromosome (Figure 2). For example, segments of chromosome 4P were also detected on the short arm of group 2 in hexaploid wheat, whereas chromosomes 2P and 7P segments were duplicated in the telomeric region of the long arm of group 4 in hexaploid wheat (Figure 2). In addition, a large number of small fragments showed obvious rearrangements throughout the P genome compared with the wheat chromosomes. For example, a small number of SNP markers on 6P belonged to the wheat homoeologous group 1A/B/D. Therefore, we inferred that this finding might reveal the generation of two T1AS·6PL·1AS·1AL intercalary translocation lines, Pubing2978 and Pubing3035, isolated from the wheat–*A. cristatum* 6P disomic substitution line 4844‐8 (Zhang *et al*., 2015a, 2016). Thus, understanding intrachromosomal rearrangements might be helpful to produce compensating translocations for gene transfer from *A. cristatum* into the wheat genome.

The comparison of marker‐based genetic maps and the gene order established in rye and wheat not only indicated well‐conserved genome collinearity but also provided evidence for multiple evolutionary translocations in the rye genome relative to that of hexaploid wheat (Bauer *et al*., 2016; Devos *et al*., 1993; Martis *et al*., 2013). For example, the long arm of 4R shows regions with homoeology to most of the short arms of wheat group 7 chromosomes, and the distal end of 4RL contains a segment with homoeology to the distal ends of the short arms of the wheat group 6 chromosomes. In addition, chromosome 6R shows homoeology with wheat groups 3 and 7 and 7R shows homoeology with wheat groups 2 and 4. The different chromosomal rearrangements between the R and P genomes indicated that species evolution was independent in rye and *Agropyron*.

### Efficient identification of P chromosome(s) in wheat–*Agropyron* derivative lines

The genetic linkage map not only enabled detailed analysis of the synteny of *Agropyron* genetic linkage maps with the wheat genome sequence (Figure 2) but also enabled the identification of wheat–*Agropyron* derivative lines (Table 1) and the mapping of individual introgressions throughout the process of backcrossing and selfing, as we could ‘tag’ introgressions using the SNP array. There has been considerable interest in exploiting the genetic variation from distant relatives of crop species for many years. However, only limited use of this enormous source of genetic variation has been applied to strategic plant breeding programmes (King *et al*., 2013). A major block to the large‐scale, genome‐wide application of genetic variation from wild relatives has been the lack of high‐throughput screening technology to quickly identify and characterize introgressions (King *et al*., 2017). The alien chromosomes in disomic addition lines can be identified by morphological analysis, chromosome banding, in situ hybridization and molecular markers (Hu *et al*., 2012; Kishii *et al*., 2004; McArthur *et al*., 2012; Schneider *et al*., 2008; Wang *et al*., 2010; Wu *et al*., 2006). In addition, *Hordeum bulbosum* introgressions in *H. vulgare* were detected using genotyping by sequencing or exome capture resequencing and mapping SNP variations to a reference genome (Wendler *et al*., 2014, 2015). In this study, we successfully used a dedicated wheat genotyping array combined with the analysis of homoeologous relationships between *Agropyron* and wheat chromosomes to identify and characterize recently developed addition/substitution lines without the need for an *A. cristatum* reference genome. Using array genotyping, we successfully screened 35 wheat–*A. cristatum* derivatives, namely 26 disomic addition lines, 3 double disomic addition lines, 5 substitution lines and 1 disomic addition and substitution line, which involves the addition of all seven P chromosomes (Table 1). The specific P genome SNP markers identified by array genotyping could conveniently be converted into KASP markers, enabling alien chromosomal segment mapping in individual introgressions. This study significantly accelerated the introgression of genetic variation from *A. cristatum* into wheat for exploitation in wheat improvement programmes.

## Experimental procedures

## Plant materials

A set of 119 individuals were obtained as the F_1_ mapping population from a cross between the two cross‐pollinated (CP) diploid collections ‘Z1842’ [*A. cristatum* (L.) Beauv., 2*n* = 2*X* = 14, PP] (male parent) and ‘Z2098’ [*A. mongolicum* Keng, 2*n* = 2*X* = 14, PP] (female parent) (Zhang *et al.,* 2015c). Wheat–*A. cristatum* alien addition/substitution lines were obtained from several generations of backcrossing or selfing following the hybridization of the common wheat Fukuho with *A. cristatum* accession ‘Z559’ (2*n* = 4*x* = 28, PPPP) (Table 1). Among these lines, II‐3‐1, II‐9‐1, II‐8‐1, II‐7‐1, II‐21‐2, II‐21‐6, 5113‐2, 5114‐3, 4844‐12, 4844‐8, 5038, 5043, II‐5‐1 and II‐26‐1 were identified previously (Han *et al*., 2017; Pan *et al*., 2017; Wu *et al*., 2006). ‘Z1842’, ‘Z2098’ and their 119 progeny, Fukuho, ‘Z559’ and addition/substitution lines were planted in the glasshouse at the Chinese Academy of Agricultural Sciences, Beijing, China. Genomic DNA was extracted from freeze‐dried young leaves using the cetyltrimethylammonium bromide (CTAB) method with minor modifications (Porebski *et al*., 1997). The components of 100 mL CTAB buffer included 2 g CTAB, 1.4 m NaCl, 20 mm EDTA, pH 8.0, and 100 mm Tris–HCl, pH 8.0. The DNA concentration and quality were estimated using a NanoDrop‐2000 spectrophotometer (Thermo Fisher Scientific, Wilmington, DE) and electrophoresis on 0.8% agarose gels with a DNA marker.

### Genotyping

**Figure 4:**
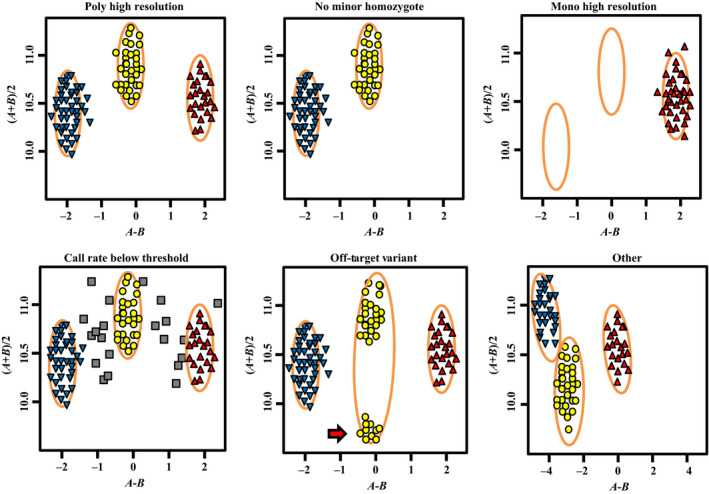
Examples of the six SNP classification categories.

The Axiom Wheat 660K Genotyping Array was used to genotype all samples described above (two parents, 119 progeny and all wheat–*A. cristatum* alien addition/substitution lines) using the Affymetrix GeneTitan System according to the manufacturer's instructions (Axiom 2.0 Assay Manual Workflow User Guide Rev3). Allele designation was performed with the Affymetrix proprietary software packages Affymetrix Power Tools (APT) and SNPolisher™ (http://www.affymetrix.com/estore/partners_programs/programs/developer/tools/devnettools.affx), and for genotyping ‘Z1842’, ‘Z2098’ and their 119 progeny, a custom software pipeline ADAP (Axiom Data Analysis Pipeline) was written in Linux bash to simplify data analysis according to the Axiom Best Practices Genotyping Workflow (http://media.affymetrix.com/support/downloads/manuals/axiom_genotyping_solution_analysis_guide.pdf). A variant call rate threshold of 80% was used instead of the default value (97%) to account for the lower call rates typically obtained from hybridizing wheat wild relatives and progenitors to the array. The apt‐probeset‐genotype program within Affymetrix Power Tools was used to determine genotype calls from Affymetrix SNP microarrays. Subsequently, the SNPolisher R package was used to calculate SNP performance metrics, such as call rate, cluster separation and deviation from expected cluster position, followed by the classification of the SNPs into performance categories. The following categories were used: (i) Poly High Resolution, representing codominant and polymorphic SNPs, with at least two examples of the minor allele; (ii) No Minor Homozygote, representing polymorphic and dominant SNPs, where with two clusters were observed; (iii) Mono High Resolution, representing monomorphic SNPs; (iv) Call Rate Below Threshold, where the SNP call rate was below the threshold but other cluster properties were above the threshold; (v) Off‐Target Variant, which included four clusters, one of which represented a null allele; and (vi) other, where one or more cluster properties were below the threshold (Figure 4).

### Linkage map construction

Polymorphisms were classified into eight segregation patterns (paternal genotype × maternal genotype): ab × cd, ef × eg, hk × hk, lm × ll, nn × np, aa × bb, ab × cc and cc × ab for the CP population. Among these patterns, only hk × hk, lm × ll and nn × np were suitable for constructing a genetic map for the F_1_ hybrid population based on the SNP array; an example of a SNP with a detailed explanation is provided in Table S8. In addition, as stated above, only the Poly High Resolution and No Minor Homozygote SNP markers were applied to construct the genetic linkage map in this study. Lep‐MAP2 software was used to filter, create LGs and order markers (Rastas *et al*., 2015). The Filtering module was used to filter markers based on high segregation distortion (dataTolerance) and excess number of missing genotypes (missingLimit) with default parameters. The SeparateChromosomes module assigns markers to LGs by computing all pairwise LOD scores between markers and joining markers with LOD scores higher than lodLimit = 11. LGs with more than 15 markers were selected for map construction. The OrderMarkers module was used to order the markers within each LG by maximizing the likelihood of the data based on the order, and Kosambi mapping was used to calculate map distances with 40 iterations. Parental genetic linkage maps (a female linkage map and a male linkage map) and an integrated linkage map were constructed. Final linkage maps were drawn using JoinMap 4.0 (Stam, 1993).

### Anchoring SNP tags into the hexaploid wheat genome

The sequence of the markers located on the current map of *Agropyron* Gaertn. was compared using BLAST (*e*‐value cut‐off of 1e‐05) against the wheat genome sequence TGACv1 (Clavijo *et al*., 2017). The homoeology and orientation of each LG were identified by obtaining the top wheat genome hits from BLAST results, and to determine orthologous map positions, the top hits were obtained for the A, B and D genomes on TGACv1 map (Clavijo *et al*., 2017). Collinearity between the P genome and the A, B and D wheat genomes was analysed by identifying collinearity blocks using the program MCScanX (Wang *et al*., 2012). To generate collinearity figures, the length of every *Agropyron* Gaertn. chromosomes was set to 500 Mbp and scaled up with cM linkage distances of marker pairs to match similar base pair lengths for the wheat genome chromosomes. Figure 2, Figures S5 and S6 were visualized using Circos v. 0.69 (Krzywinski *et al*., 2009) to observe collinearity between *Agropyron* Gaertn. and the A, B and D wheat genomes.

### GISH analysis

Thirty randomly selected plants were used for GISH analysis to determine the chromosome composition in each line. Wheat Fukuho genomic DNA was used as blocking DNA, and the entire *A. cristatum* ‘Z559’ genome was used as a probe to detect the P genome chromosome. Chromosomes prepared from the plant root tip cells were used for GISH analysis according to Cuadrado *et al*. (2000) and the improved procedure described by Liu *et al*. (2010). Three to five cells per plant were analysed using a BX51 Olympus phase‐contrast microscope (Olympus Corp., Tokyo, Japan), and in situ hybridization images were obtained using an Olympus AX80 (Japan) fluorescence microscope and processed using Photoshop CS 3.0 (Adobe, San Jose, CA).

### Identification of addition/substitution lines

All addition/substitution lines were also genotyped using the Axiom Wheat 660K Genotyping Arrays according to the procedure stated above. In theory, with the homozygous wheat genome background, the proportion of heterozygous genotypes on wheat chromosomes that are homoeologous to introgressed alien chromosomes should be higher than for other alien chromosomes, and the substituted wheat chromosomes will have more missed genotype markers than other wheat chromosomes. Thus, the ratio of the heterozygous genotypes on each of the wheat chromosomes for each addition/substitution lines was determined, and the P chromosomes that were homoeologous to the highest proportion of wheat chromosomes were considered additional alien chromosomes. The proportion of missed genotypes in wheat each chromosome for every substitution line was also counted, and the chromosome with the highest missing genotype is the replaced wheat chromosome in the substitution lines. The A/B/D genome‐specific SNP markers were used only to identify the missing wheat chromosomes.

## Supporting information


**Figure S1** Distribution of samples and SNPs according to call rates in the wheat 660K SNP assay.

**Figure S2** The male genetic linkage map.

**Figure S3** The female genetic linkage map.

**Figure S4** Syntenic relationship between the integrated map and parental maps.

**Figure S5** Synteny of the male map (genetic position in cM) with hexaploid wheat (physical position in Mb).

**Figure S6** Synteny of the female map (genetic position in cM) with hexaploid wheat (physical position in Mb).

**Figure S7** GISH analysis of partial derivatives.

**Figure S8** Distribution of heterozygosity in wheat genome.

**Table S1** Summary of six SNP categories

**Table S2** Summary of the three segregation patterns appropriate for the CP population

**Table S3** Basic characteristics of the male and female maps

**Table S4** Basic characteristics of the seven LGs from the integrated map

**Table S5** Distribution of SNP markers in LGs in the wheat genome

**Table S6** Summary of the SNP marker distribution for LGs in the wheat genome.

**Table S7** Statistical analysis of missing genotypes of each wheat chromosome in each substitution line to identify missing wheat chromosomes

**Table S8** An example of the segregation patterns of a SNP with 4 alleles (A/T/C/G)
