# Supplementary material for: Construction of Agropyron Gaertn. genetic linkage maps using a wheat 660K SNP array reveals a homoeologous relationship with the wheat genome
Source: Plant Biotechnol J. 2017 Oct 16;16(3):818–27. doi: 10.1111/pbi.12831 (PMC5814592; doi:10.1111/pbi.12831)
Supplement: Supplementary file 1 — Figure S1 Distribution of samples and SNPs according to call rates in the wheat 660K SNP assay. [file PBI-16-818-s016.pptx]

## Slide 1
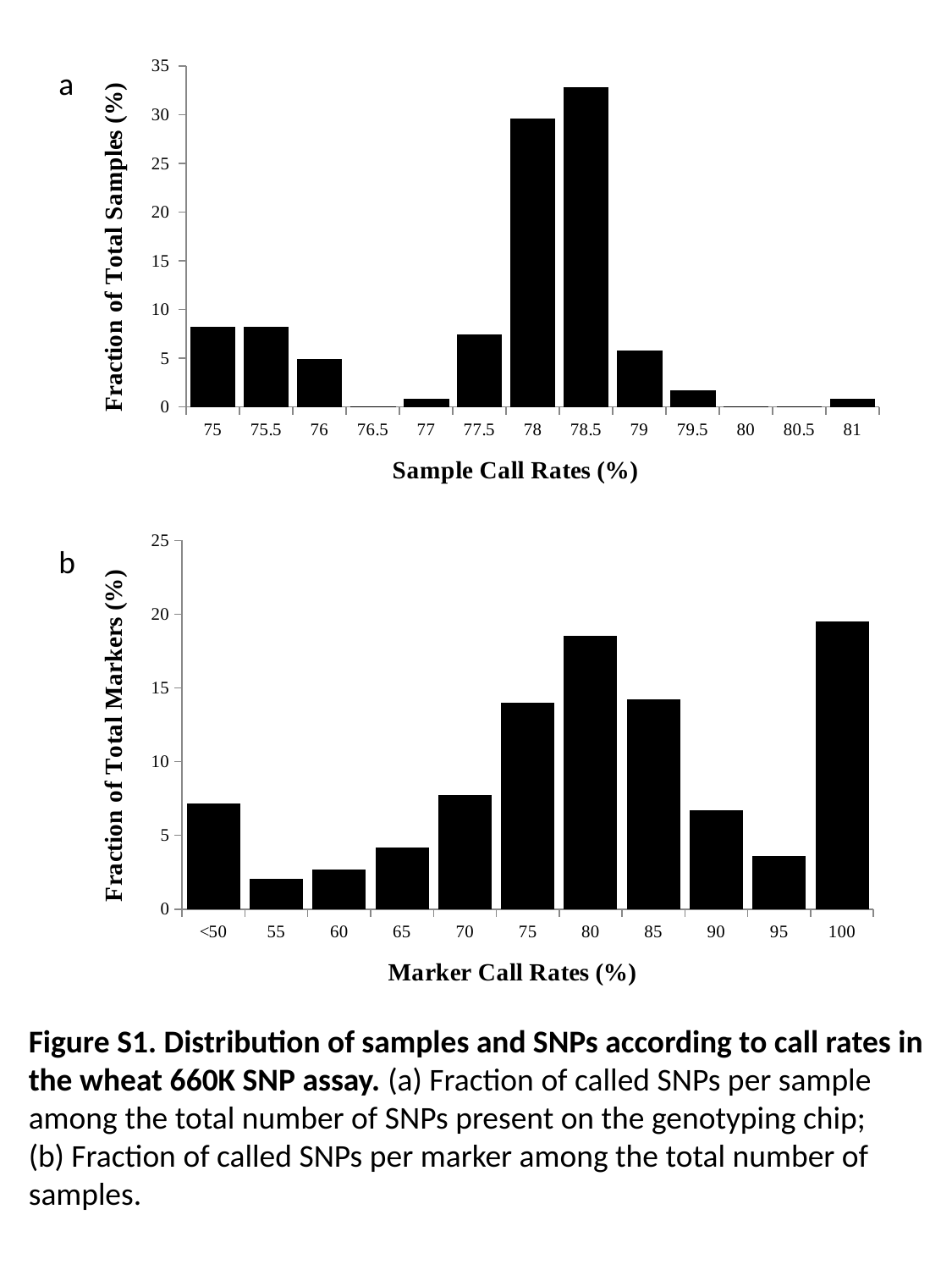

### Chart
| Category | |
|---|---|
| 75 | 8.19672131147541 |
| 75.5 | 8.19672131147541 |
| 76 | 4.918032786885246 |
| 76.5 | 0.0 |
| 77 | 0.819672131147541 |
| 77.5 | 7.377049180327869 |
| 78 | 29.508196721311474 |
| 78.5 | 32.78688524590164 |
| 79 | 5.737704918032787 |
| 79.5 | 1.639344262295082 |
| 80 | 0.0 |
| 80.5 | 0.0 |
| 81 | 0.819672131147541 |a
### Chart
| Category | |
|---|---|
| <50 | 7.109245270151321 |
| 55 | 1.9991530759678169 |
| 60 | 2.6695553014431015 |
| 65 | 4.128675356889663 |
| 70 | 7.682584291938204 |
| 75 | 13.959655330466902 |
| 80 | 18.525115103954374 |
| 85 | 14.21151214003746 |
| 90 | 6.655649252914672 |
| 95 | 3.596413736663722 |
| 100 | 19.462441139572764 |b
Figure S1. Distribution of samples and SNPs according to call rates in the wheat 660K SNP assay. (a) Fraction of called SNPs per sample among the total number of SNPs present on the genotyping chip;
(b) Fraction of called SNPs per marker among the total number of samples.
