# Supplementary figures and images for: Construction of Agropyron Gaertn. genetic linkage maps using a wheat 660K SNP array reveals a homoeologous relationship with the wheat genome
Source: Plant Biotechnol J. 2017 Oct 16;16(3):818–27. doi: 10.1111/pbi.12831 (PMC5814592; doi:10.1111/pbi.12831)

## Slide 1
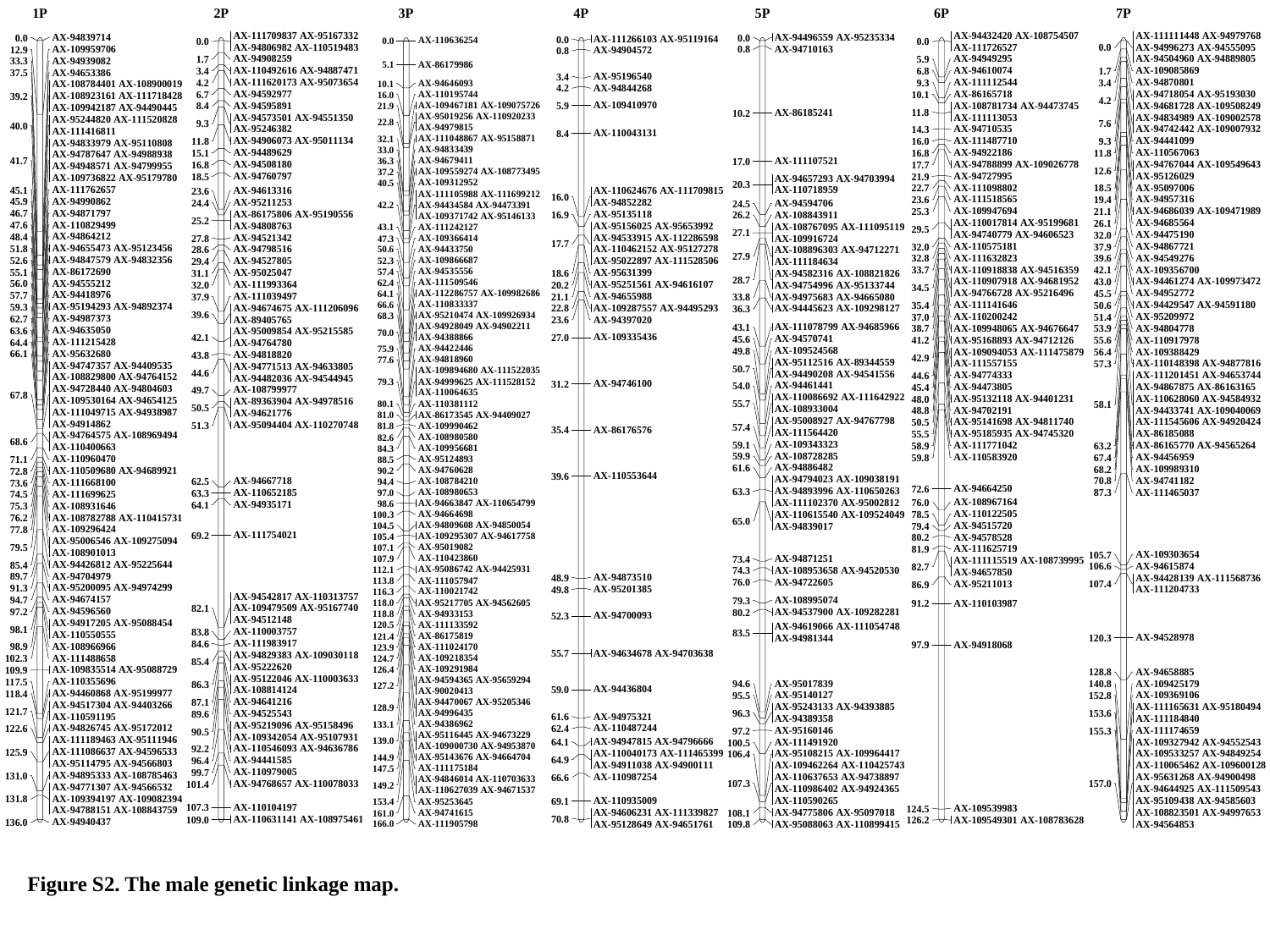

Figure S2. The male genetic linkage map.

Supplement: Supplementary file 2 — Figure S2 The male genetic linkage map. [file PBI-16-818-s015.pptx]

## Slide 1
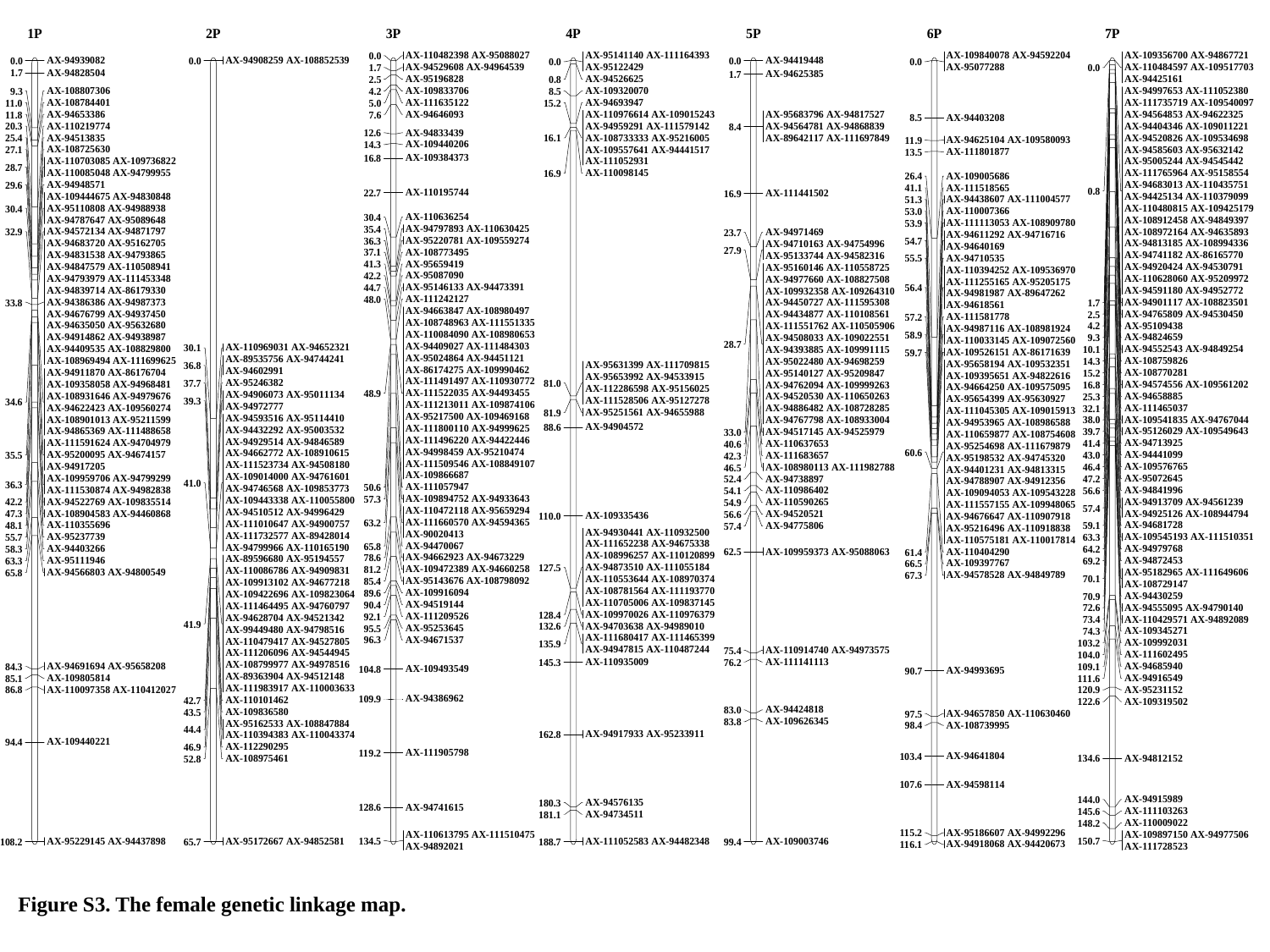

Figure S3. The female genetic linkage map.

Supplement: Supplementary file 3 — Figure S3 The female genetic linkage map. [file PBI-16-818-s014.pptx]
