# Supplementary material for: Construction of Agropyron Gaertn. genetic linkage maps using a wheat 660K SNP array reveals a homoeologous relationship with the wheat genome
Source: Plant Biotechnol J. 2017 Oct 16;16(3):818–27. doi: 10.1111/pbi.12831 (PMC5814592; doi:10.1111/pbi.12831)
Supplement: Supplementary file 4 — Figure S4 Syntenic relationship between the integrated map and parental maps. [file PBI-16-818-s013.pptx]

## Slide 1
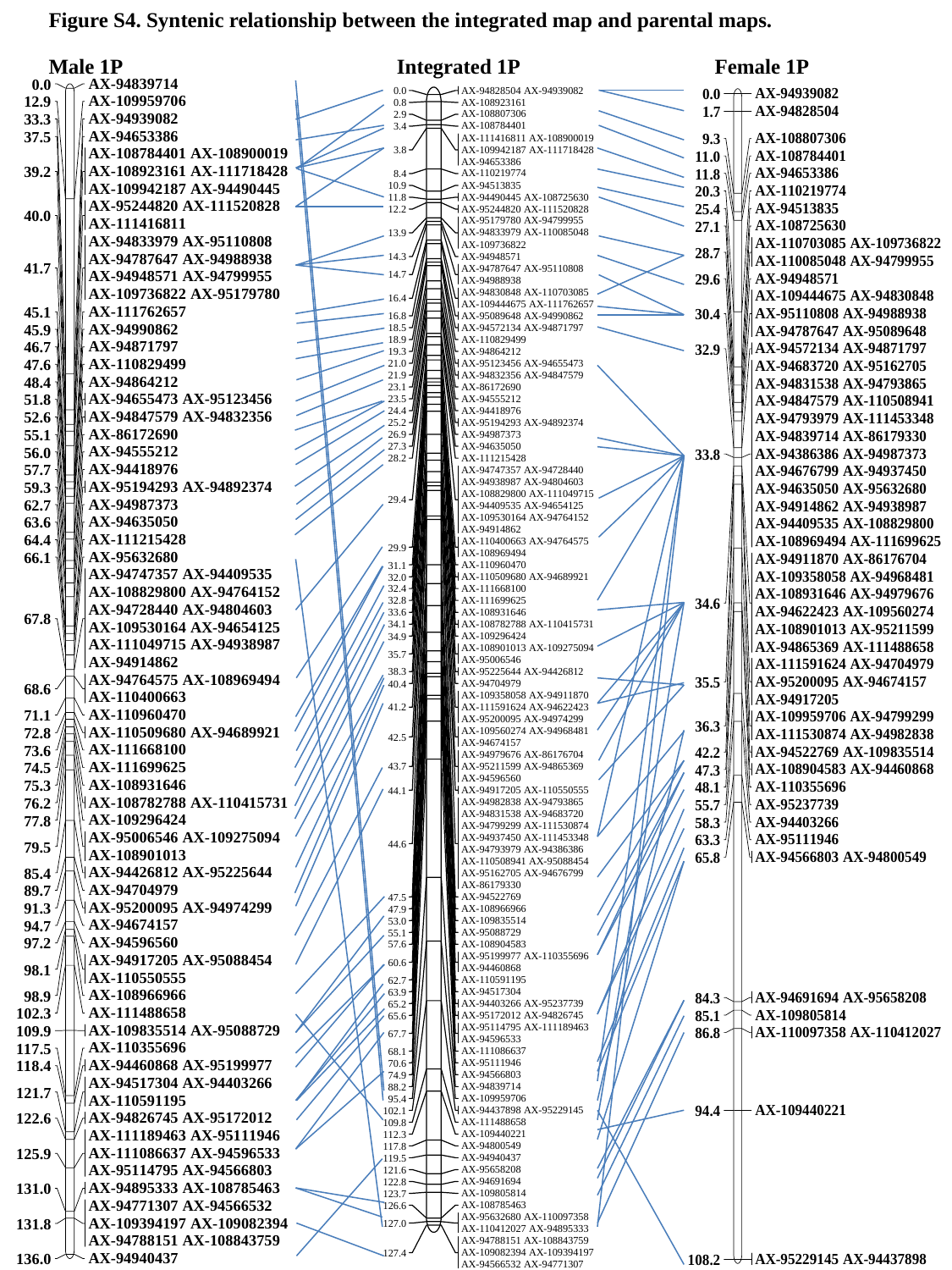

Figure S4. Syntenic relationship between the integrated map and parental maps.
Male 1P Integrated 1P Female 1P

## Slide 2
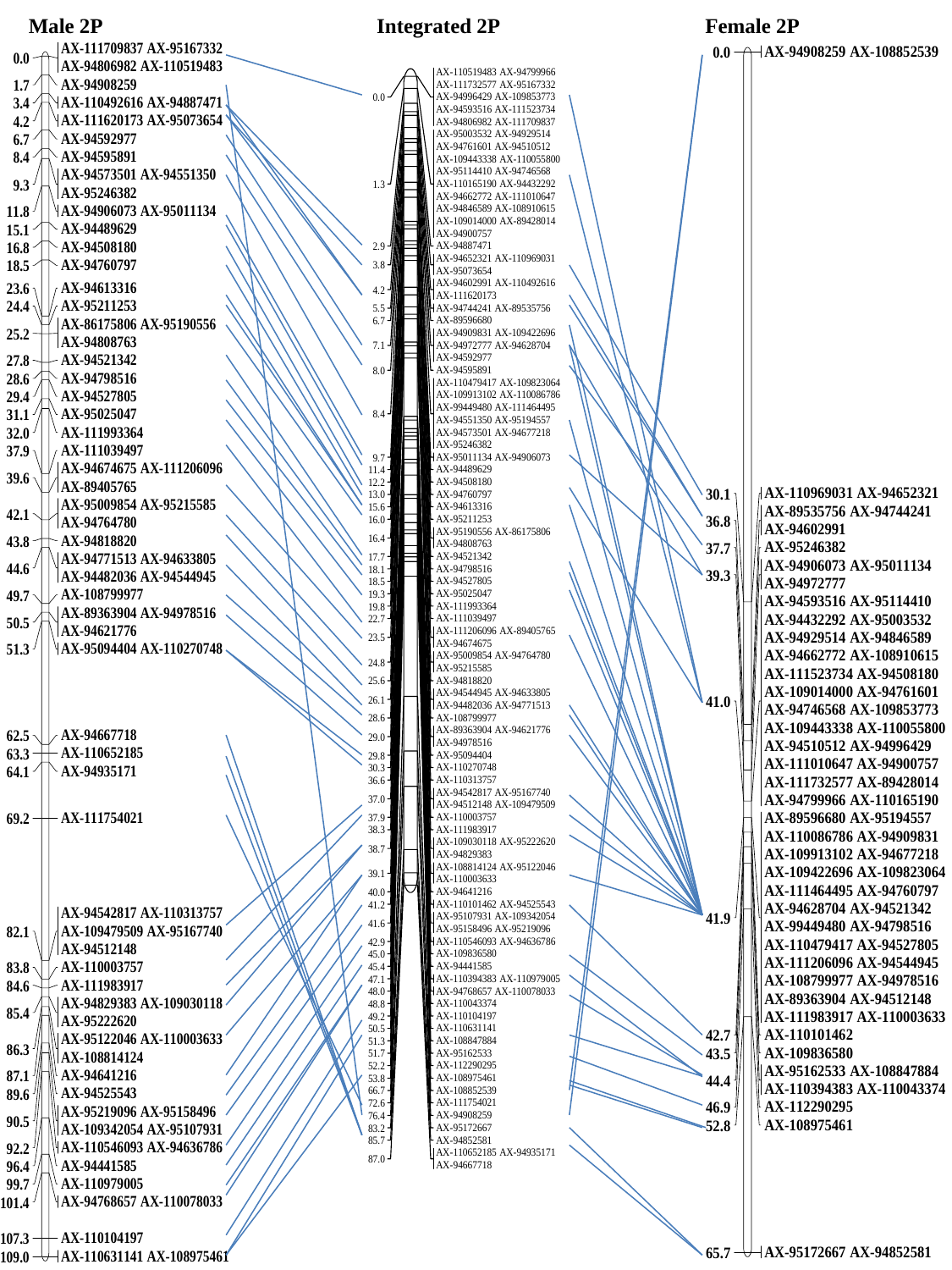

Male 2P Integrated 2P Female 2P

## Slide 3
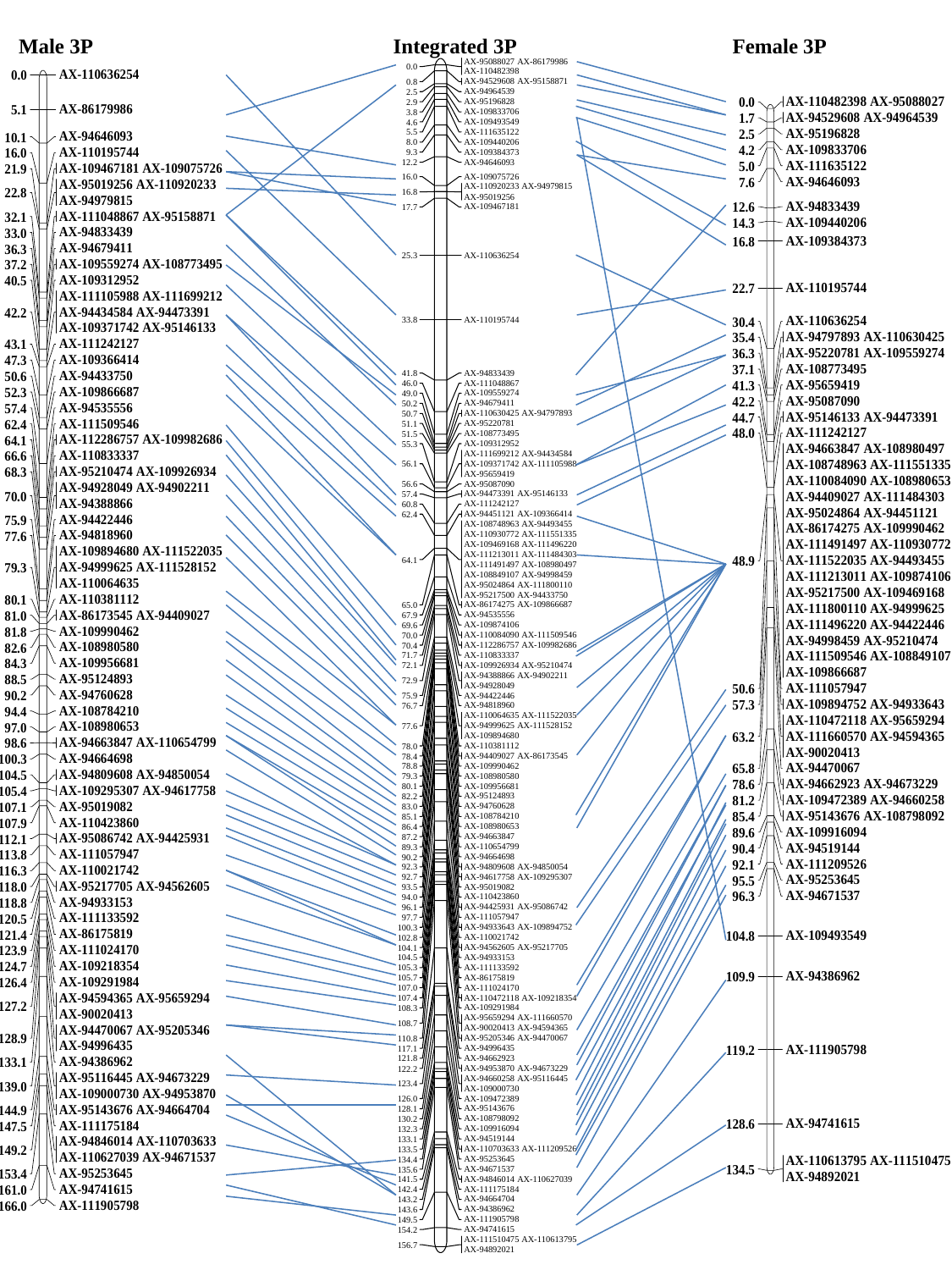

Male 3P Integrated 3P Female 3P

## Slide 4
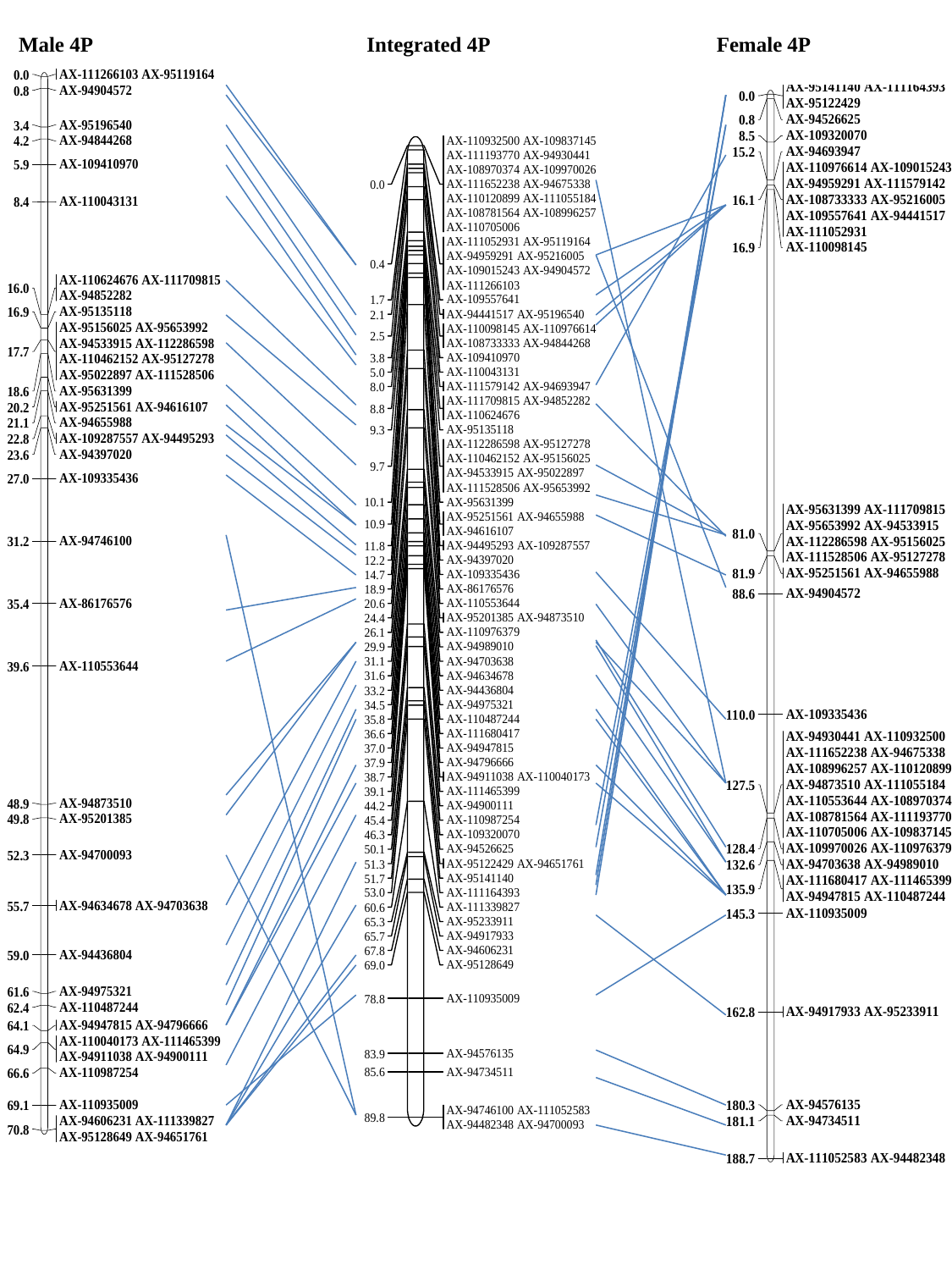

Male 4P Integrated 4P Female 4P

## Slide 5
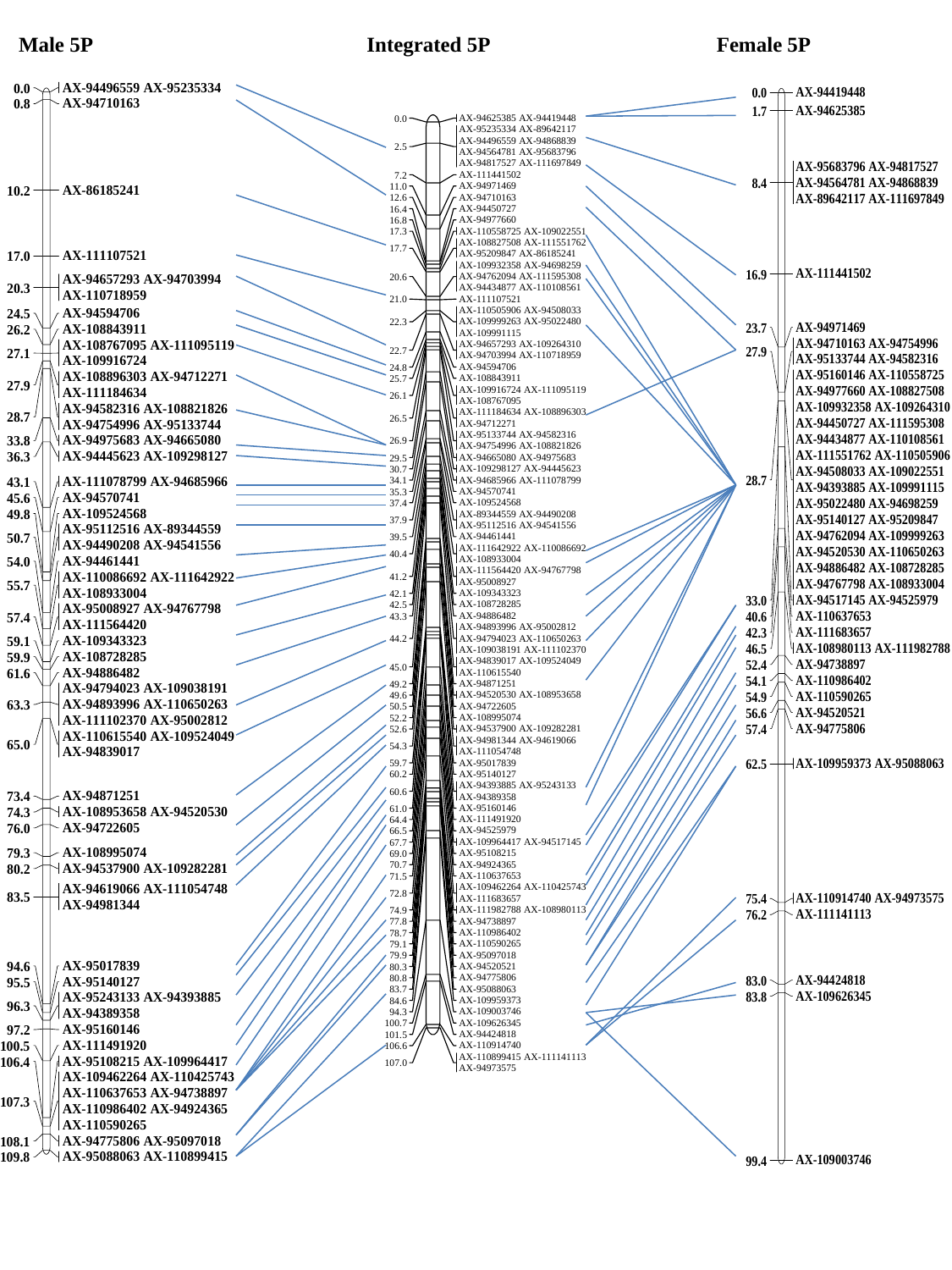

Male 5P Integrated 5P Female 5P

## Slide 6
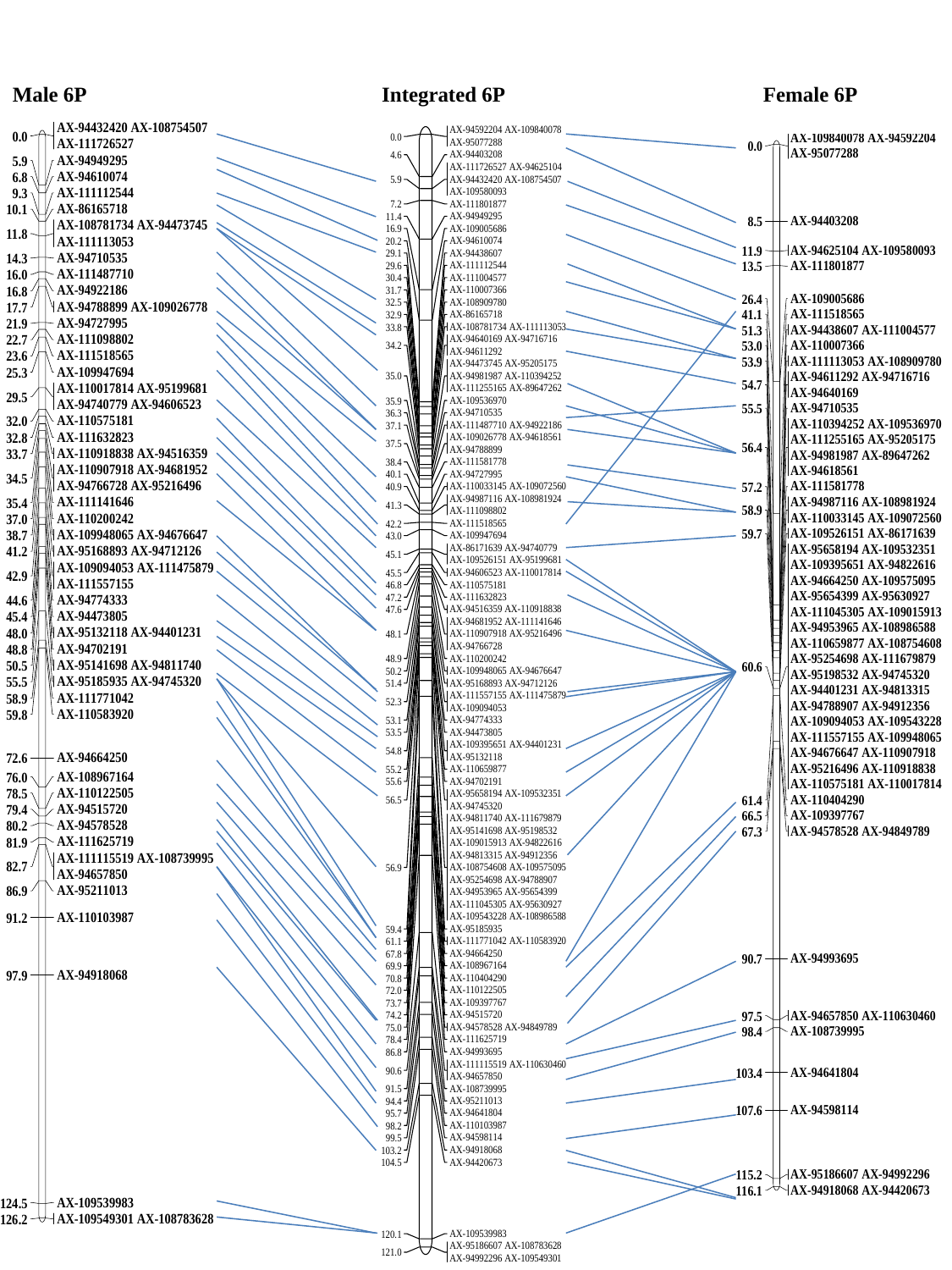

Male 6P Integrated 6P Female 6P

## Slide 7
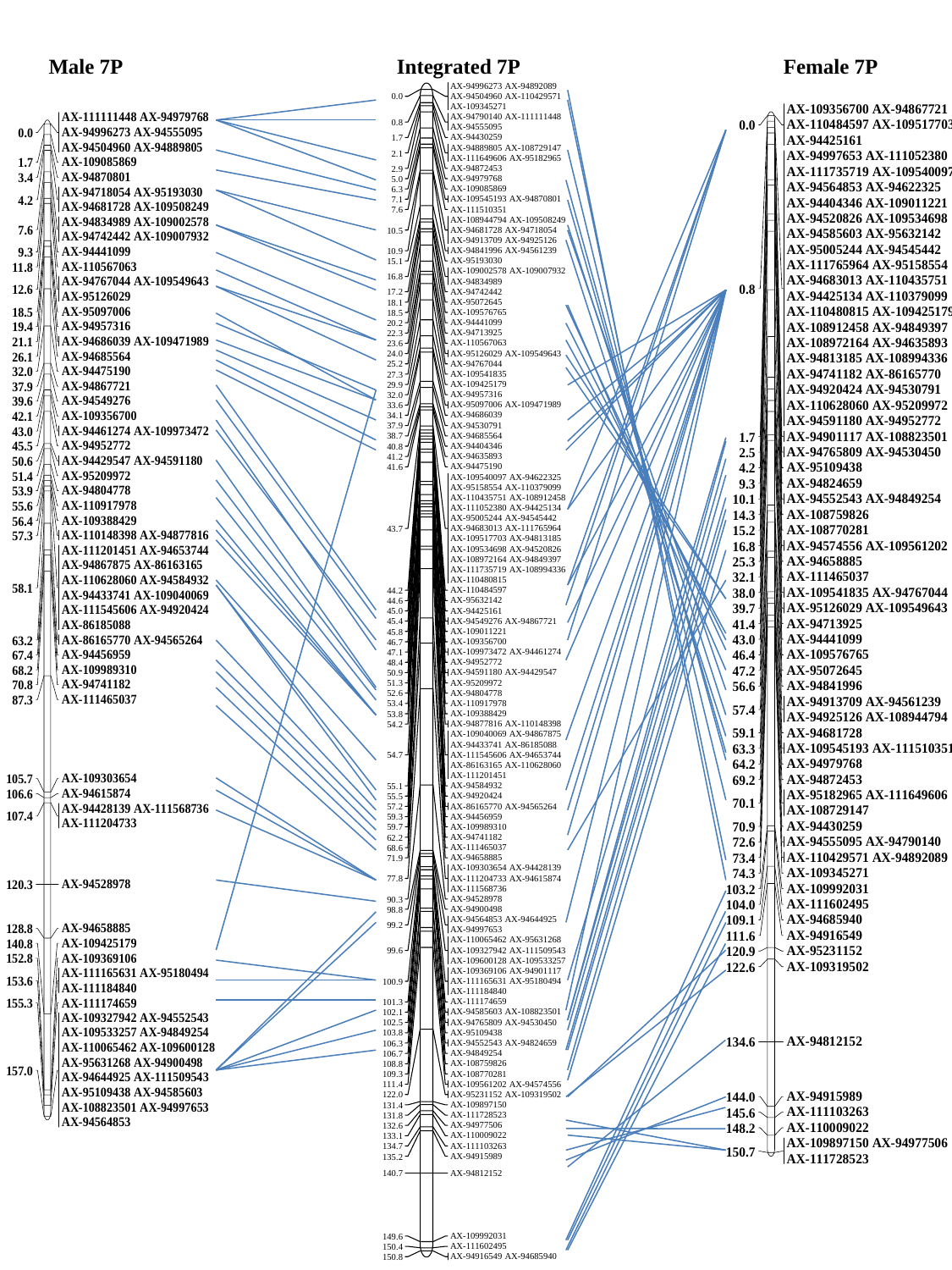

Male 7P Integrated 7P Female 7P
