# Supplementary material for: Construction of Agropyron Gaertn. genetic linkage maps using a wheat 660K SNP array reveals a homoeologous relationship with the wheat genome
Source: Plant Biotechnol J. 2017 Oct 16;16(3):818–27. doi: 10.1111/pbi.12831 (PMC5814592; doi:10.1111/pbi.12831)
Supplement: Supplementary file 6 — Figure S6 Synteny of the female map (genetic position in cM) with hexaploid wheat (physical position in Mb). [file PBI-16-818-s011.pptx]

## Slide 1
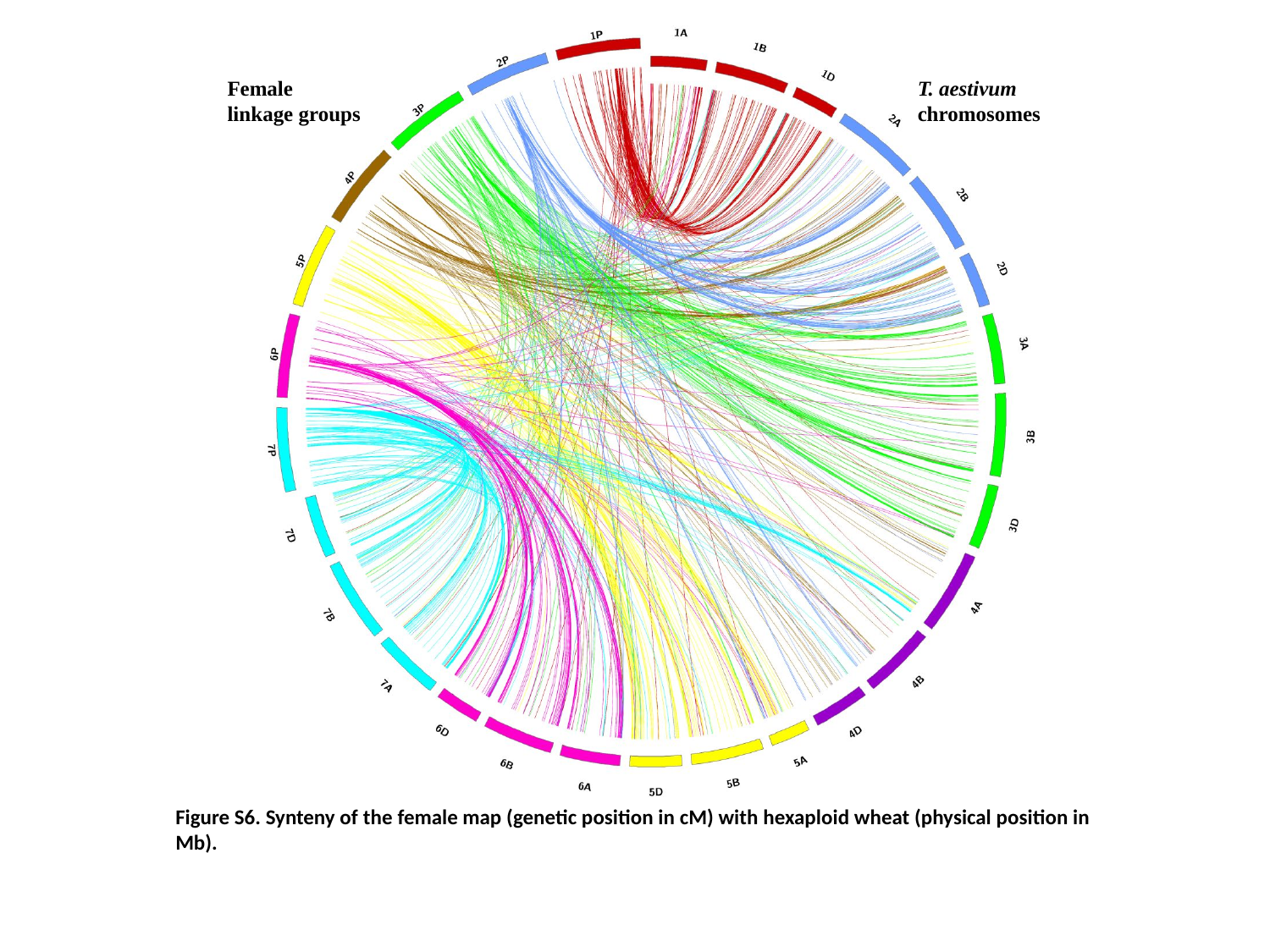

Female
linkage groups
T. aestivum
chromosomes
Figure S6. Synteny of the female map (genetic position in cM) with hexaploid wheat (physical position in Mb).
