# Supplementary material for: Construction of Agropyron Gaertn. genetic linkage maps using a wheat 660K SNP array reveals a homoeologous relationship with the wheat genome
Source: Plant Biotechnol J. 2017 Oct 16;16(3):818–27. doi: 10.1111/pbi.12831 (PMC5814592; doi:10.1111/pbi.12831)
Supplement: Supplementary file 7 — Figure S7 GISH analysis of partial derivatives. [file PBI-16-818-s001.pptx]

## Slide 1
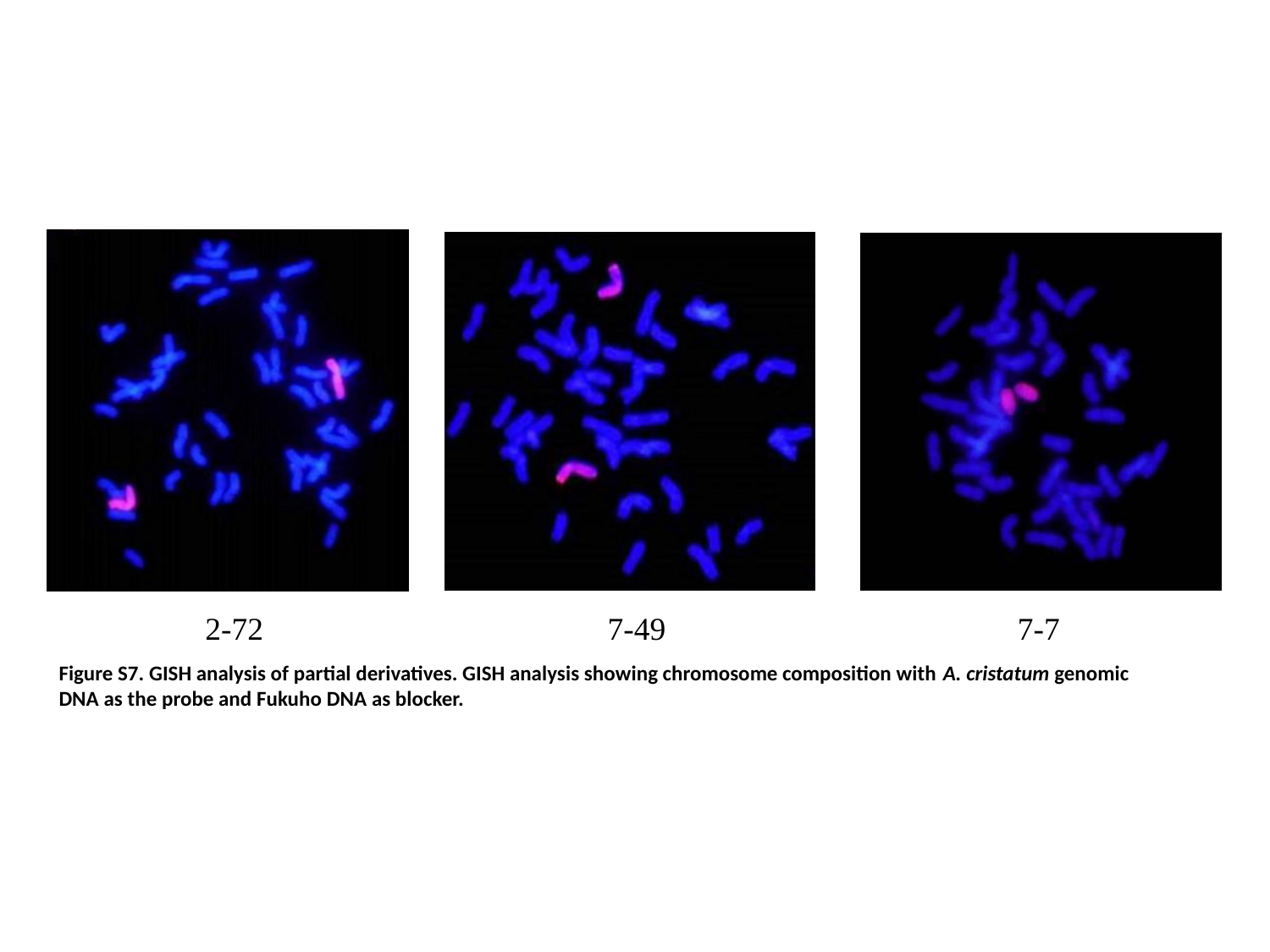

2-72
7-49
7-7
Figure S7. GISH analysis of partial derivatives. GISH analysis showing chromosome composition with A. cristatum genomic DNA as the probe and Fukuho DNA as blocker.
