# Supplementary material for: Construction of Agropyron Gaertn. genetic linkage maps using a wheat 660K SNP array reveals a homoeologous relationship with the wheat genome
Source: Plant Biotechnol J. 2017 Oct 16;16(3):818–27. doi: 10.1111/pbi.12831 (PMC5814592; doi:10.1111/pbi.12831)
Supplement: Supplementary file 8 — Figure S8 Distribution of heterozygosity in wheat genome. [file PBI-16-818-s002.pptx]

## Slide 1
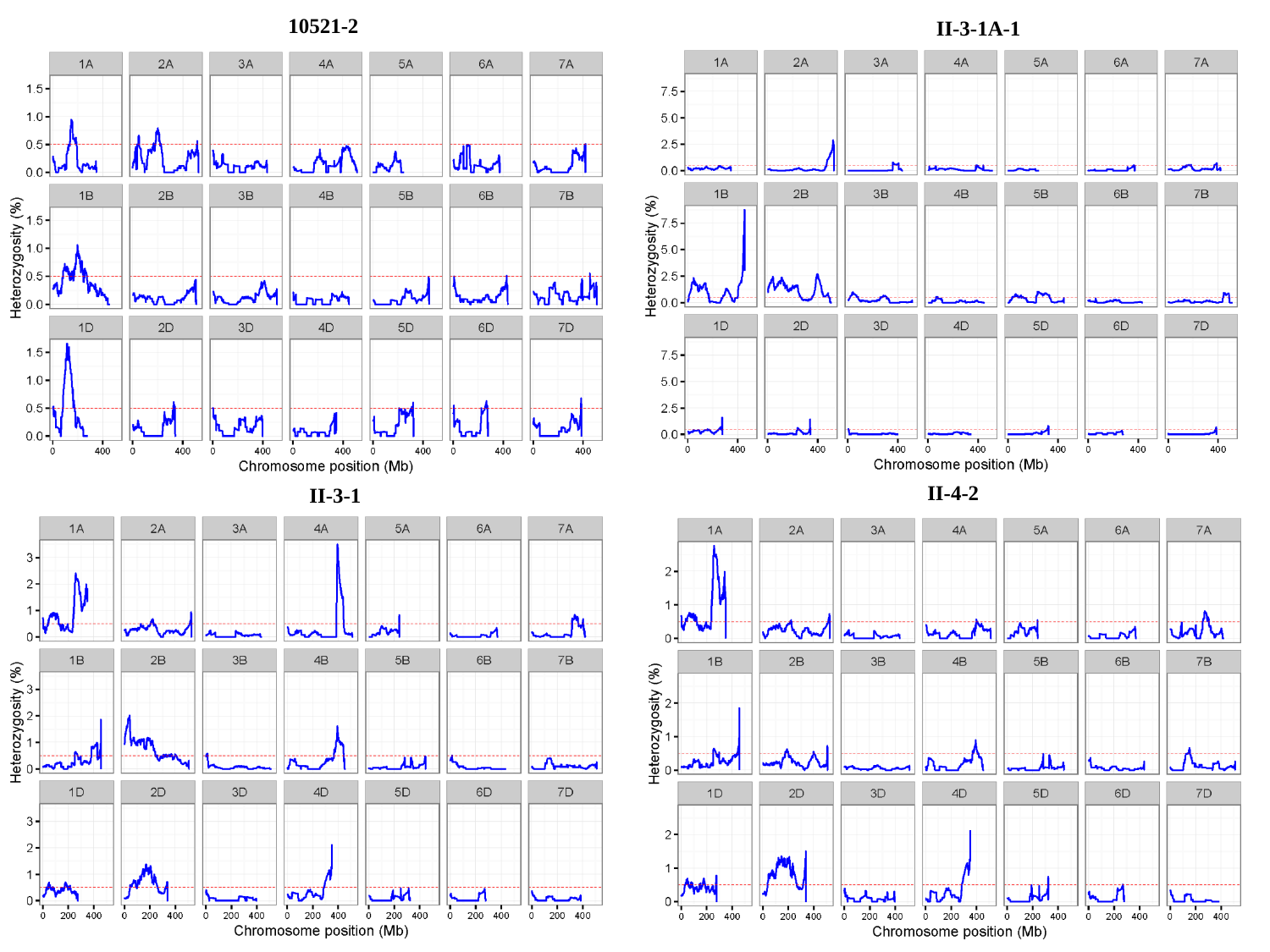

10521-2
II-3-1A-1
II-4-2
II-3-1

## Slide 2
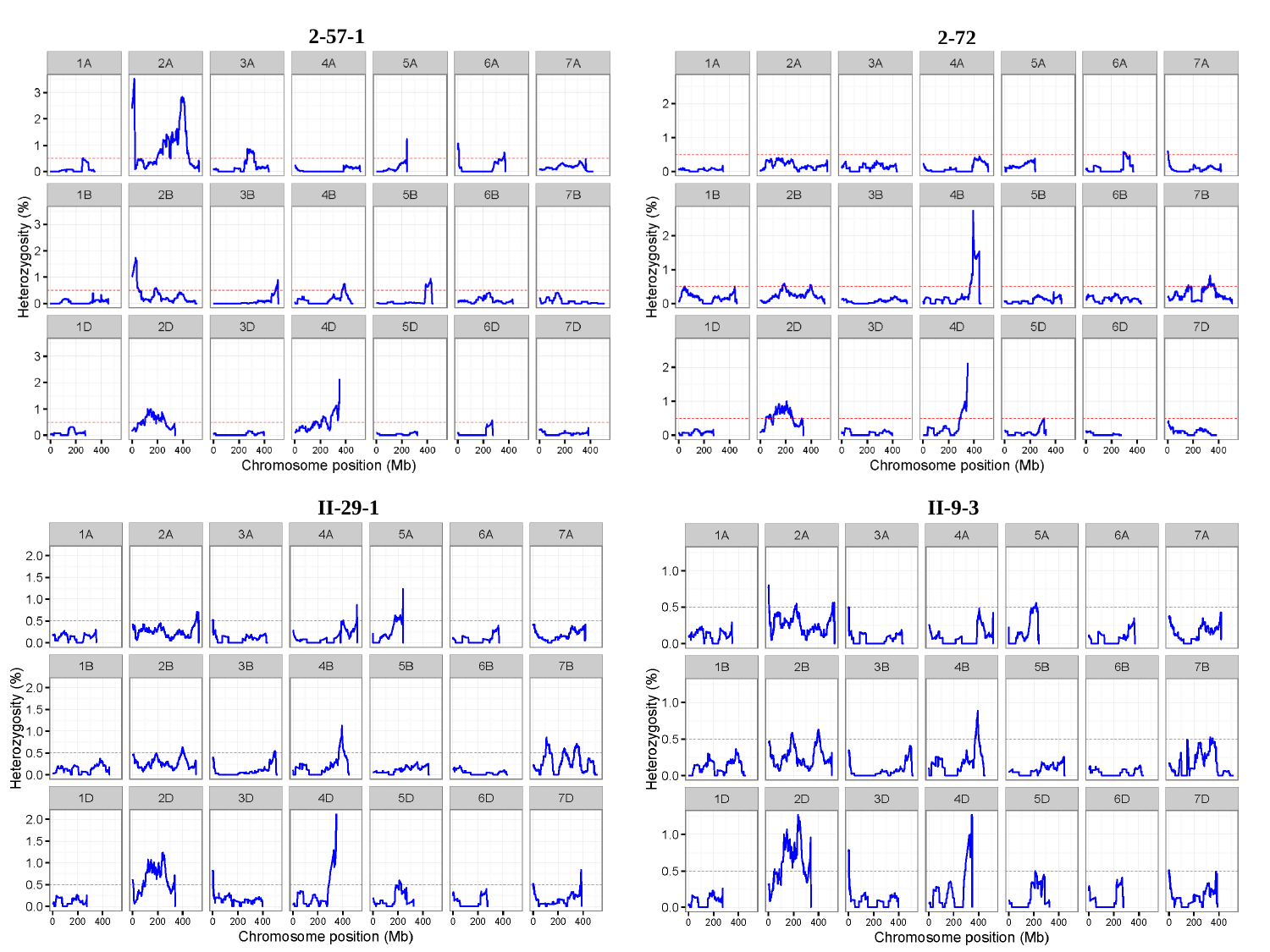

2-57-1
2-72
II-29-1
II-9-3

## Slide 3
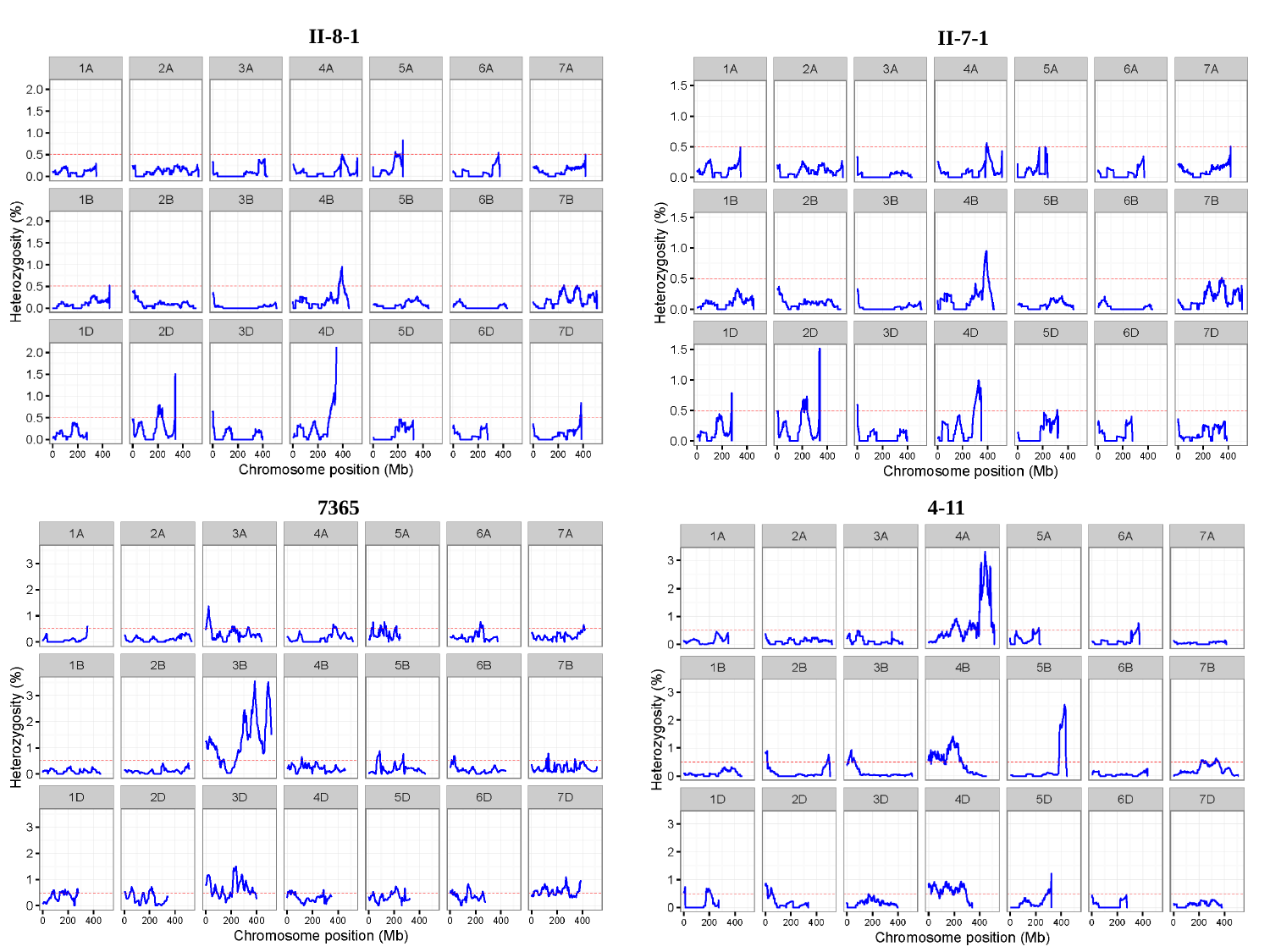

II-8-1
II-7-1
7365
4-11

## Slide 4
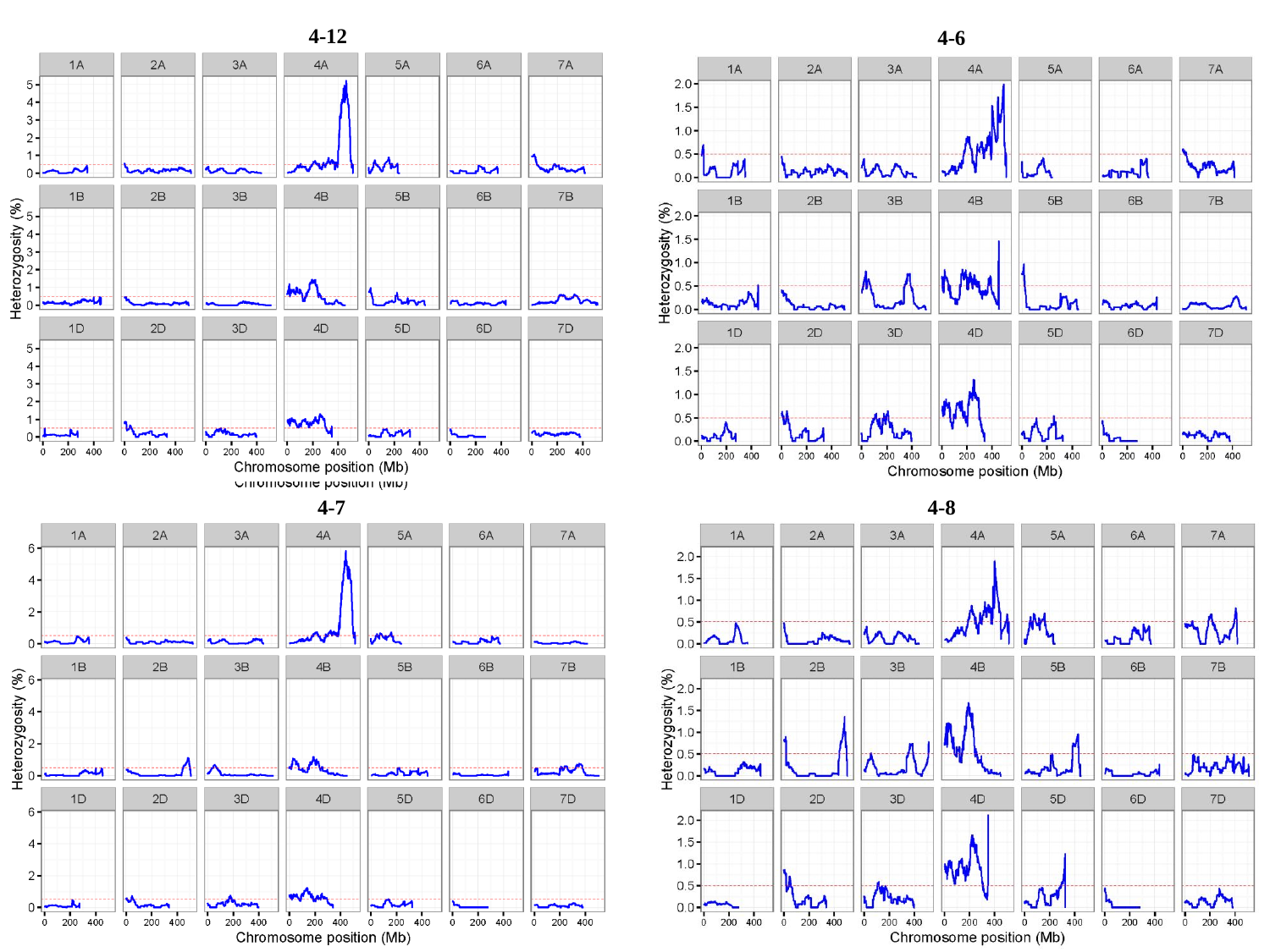

4-12
4-6
4-7
4-8

## Slide 5
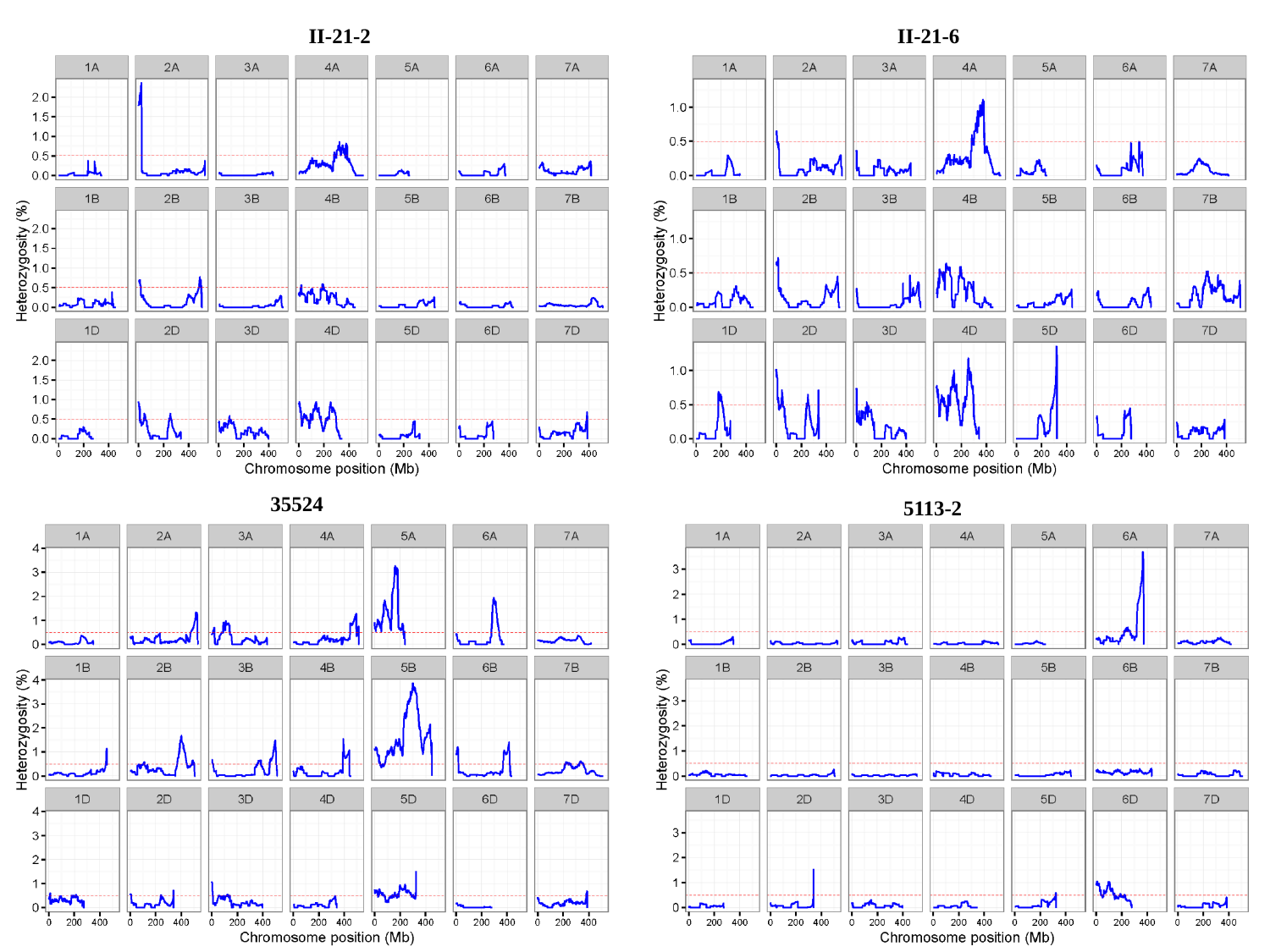

II-21-6
II-21-2
35524
5113-2

## Slide 6
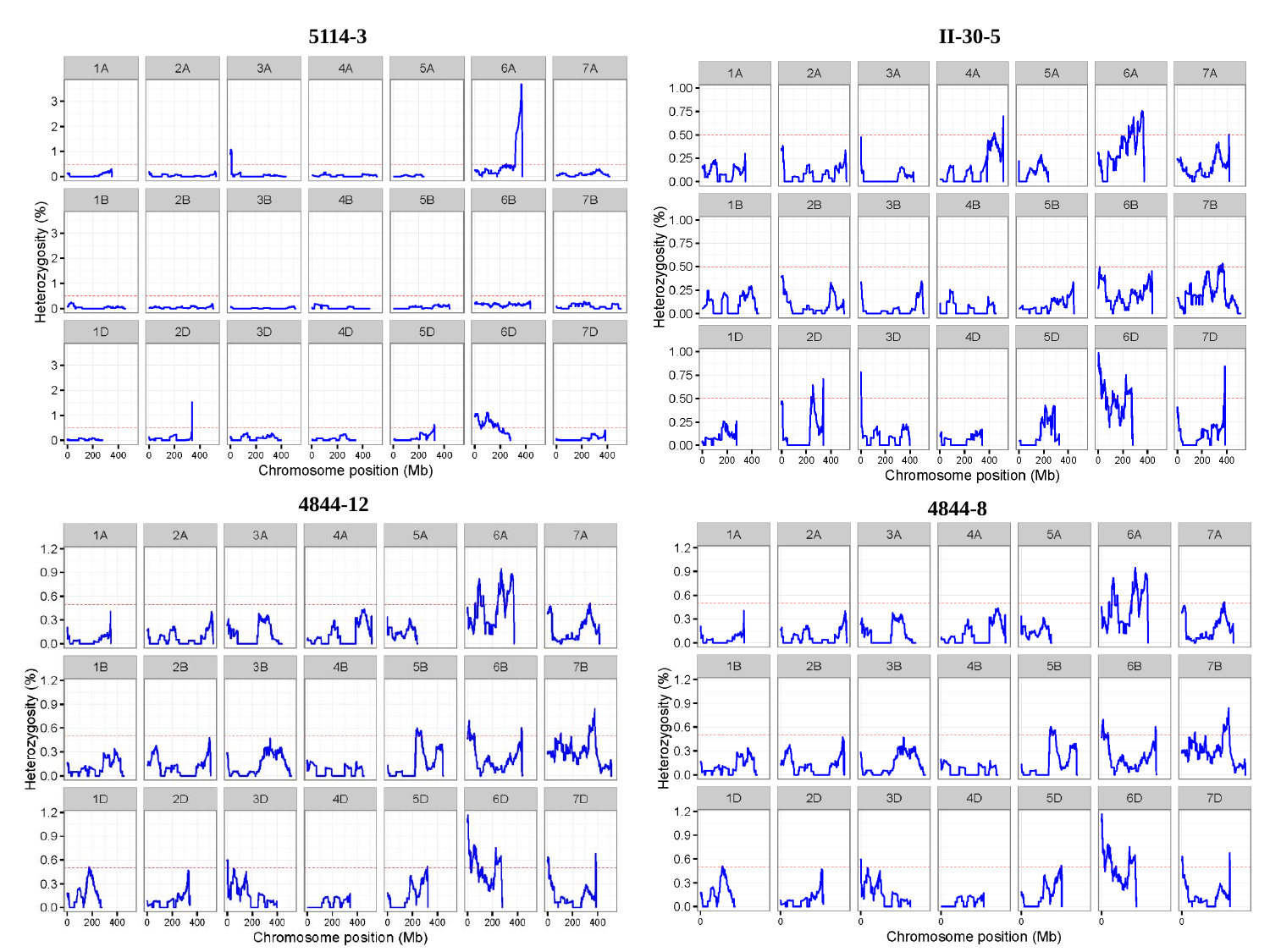

II-30-5
5114-3
4844-12
4844-8

## Slide 7
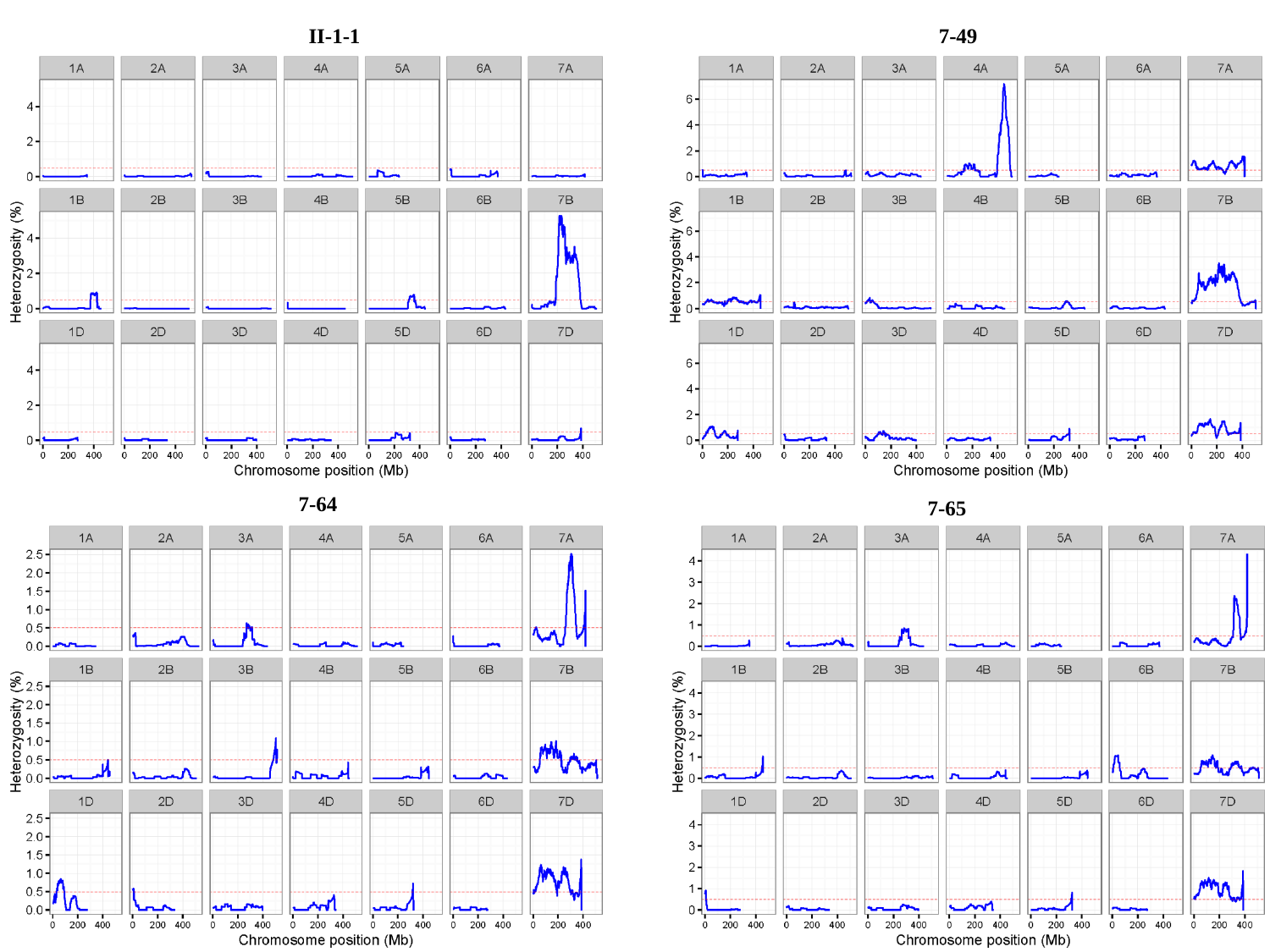

7-49
II-1-1
7-64
7-65

## Slide 8
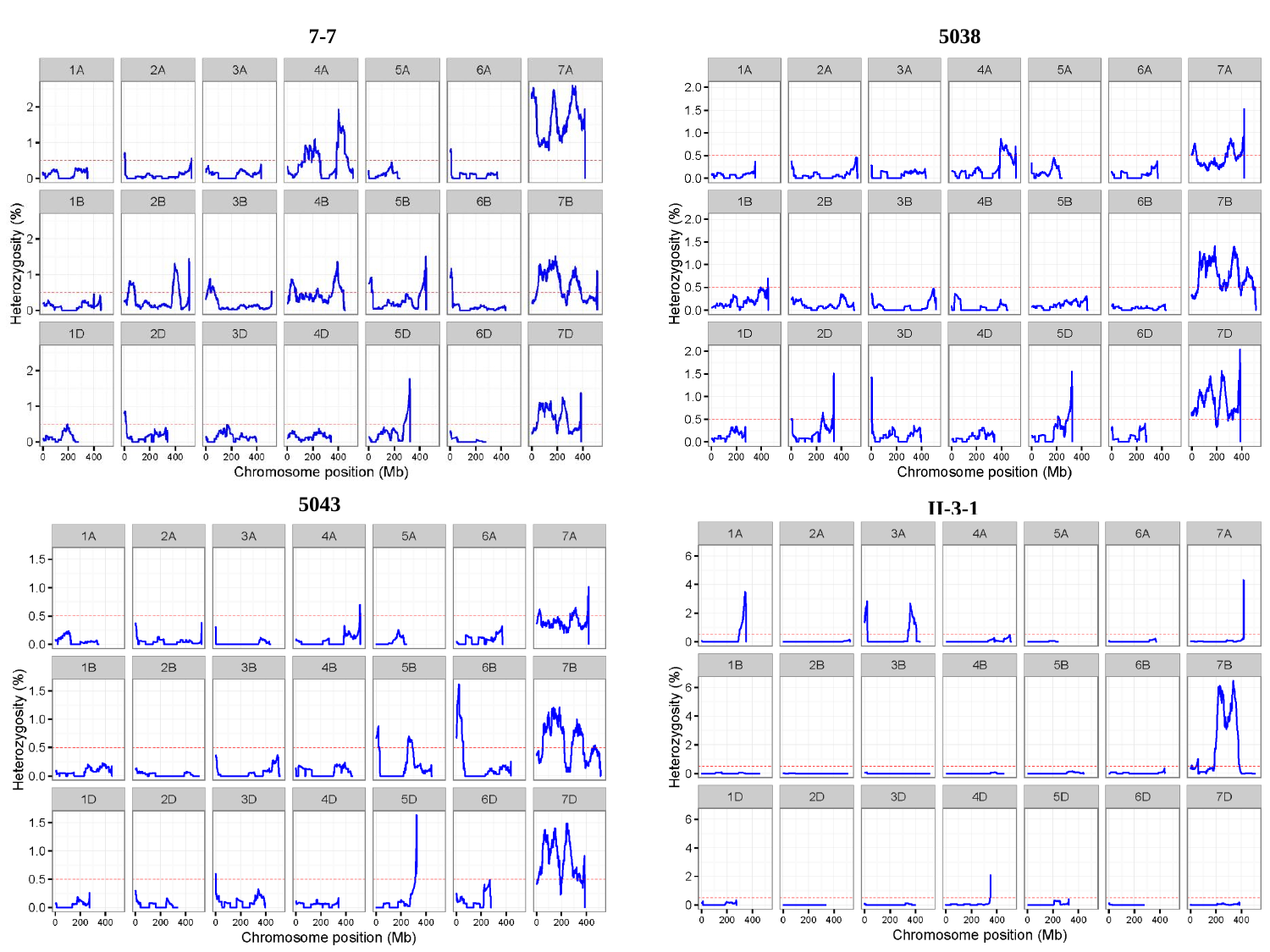

5038
7-7
5043
II-3-1

## Slide 9
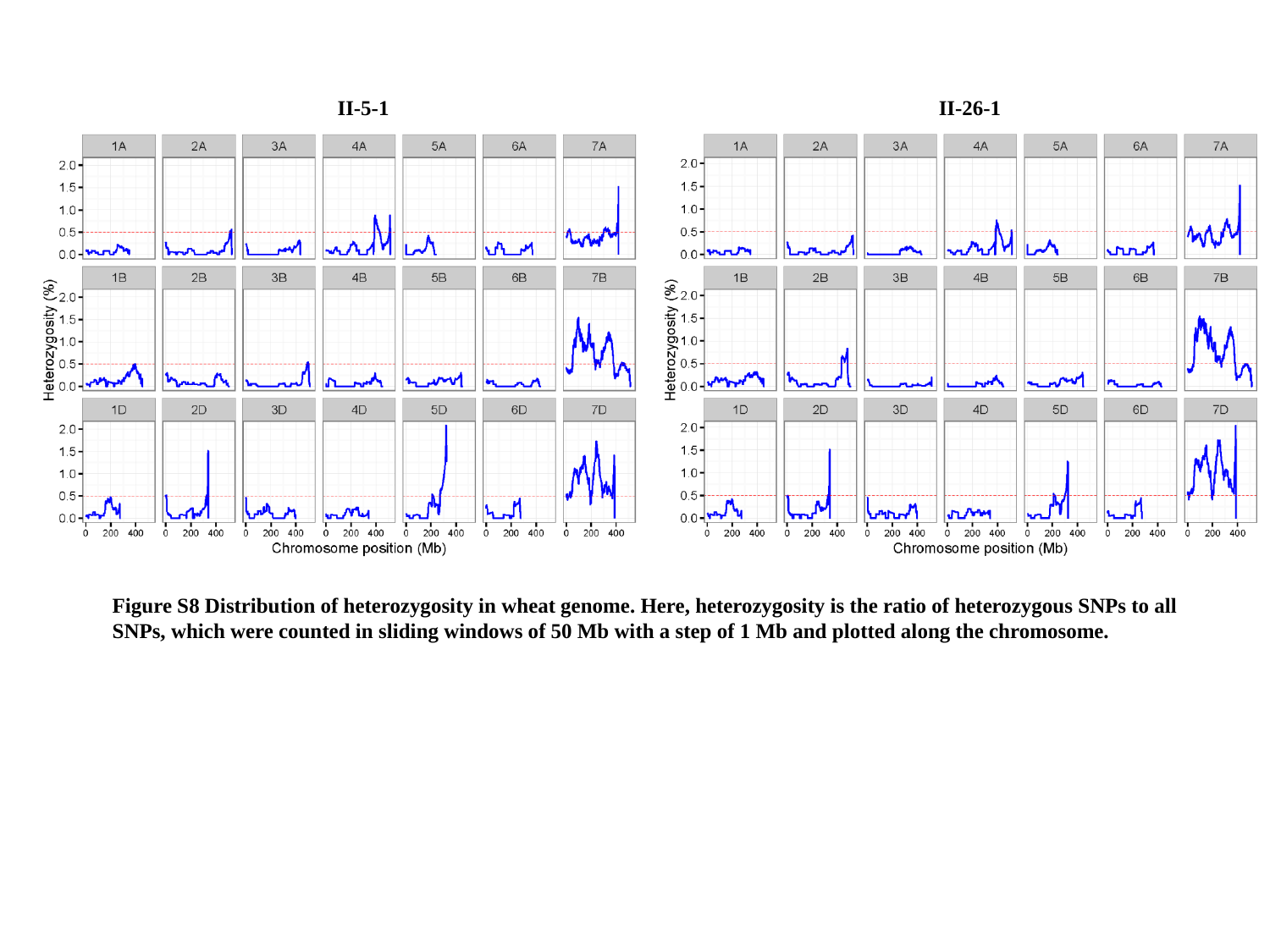

II-26-1
II-5-1
Figure S8 Distribution of heterozygosity in wheat genome. Here, heterozygosity is the ratio of heterozygous SNPs to all SNPs, which were counted in sliding windows of 50 Mb with a step of 1 Mb and plotted along the chromosome.
